# Tuning the Cloud-Point and Flocculation Temperature of Poly(2-(diethylamino)ethyl methacrylate)-Based Nanoparticles via a Postpolymerization Betainization Approach

**DOI:** 10.1021/acspolymersau.1c00010

**Published:** 2021-07-08

**Authors:** Matthieu P. J. Miclotte, Stefan B. Lawrenson, Spyridon Varlas, Bilal Rashid, Emma Chapman, Rachel K. O’Reilly

**Affiliations:** † School of Chemistry, University of Birmingham, Edgbaston, Birmingham B15 2TT, United Kingdom; ‡ BP Exploration Operating Company Ltd., Chertsey Road, Sunbury-on-Thames, Middlesex TW16 7LN, United Kingdom

**Keywords:** thermoresponsive, critical solution temperature, PDEAEMA, betainization, emulsion polymerization

## Abstract

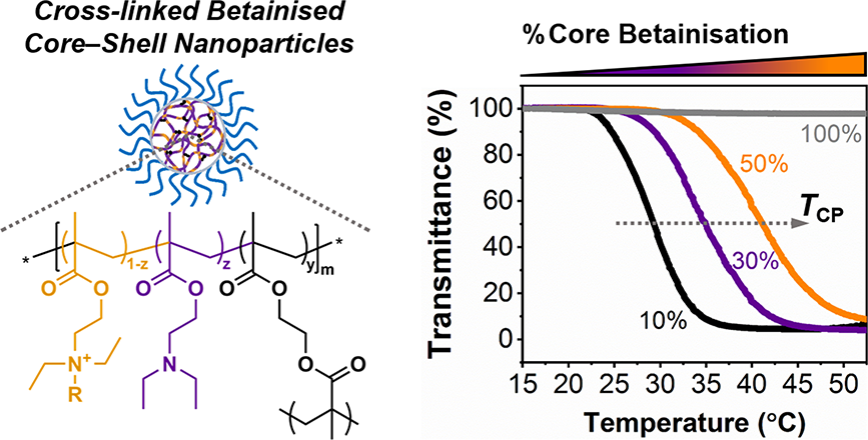

The ability to tune the behavior of temperature-responsive polymers and self-assembled nanostructures has attracted significant interest in recent years, particularly in regard to their use in biotechnological applications. Herein, well-defined poly(2-(diethylamino)ethyl methacrylate) (PDEAEMA)-based core–shell particles were prepared by RAFT-mediated emulsion polymerization, which displayed a lower-critical solution temperature (LCST) phase transition in aqueous media. The tertiary amine groups of PDEAEMA units were then utilized as functional handles to modify the core-forming block chemistry via a postpolymerization betainization approach for tuning both the cloud-point temperature (*T*_CP_) and flocculation temperature (*T*_CFT_) of these particles. In particular, four different sulfonate salts were explored aiming to investigate the effect of the carbon chain length and the presence of hydroxyl functionalities alongside the carbon spacer on the particle’s thermoresponsiveness. In all cases, it was possible to regulate both *T*_CP_ and *T*_CFT_ of these nanoparticles upon varying the degree of betainization. Although *T*_CP_ was found to be dependent on the type of betainization reagent utilized, it only significantly increased for particles betainized using sodium 3-chloro-2-hydroxy-1-propanesulfonate, while varying the aliphatic chain length of the sulfobetaine only provided limited temperature variation. In comparison, the onset of flocculation for betainized particles varied over a much broader temperature range when varying the degree of betainization with no real correlation identified between *T*_CFT_ and the sulfobetaine structure. Moreover, experimental results were shown to partially correlate to computational oligomer hydrophobicity calculations. Overall, the innovative postpolymerization betainization approach utilizing various sulfonate salts reported herein provides a straightforward methodology for modifying the thermoresponsive behavior of soft polymeric particles with potential applications in drug delivery, sensing, and oil/lubricant viscosity modification.

## Introduction

Stimuli-responsive (or “smart”) polymers exhibit a change in their physical and/or chemical properties in response to an externally applied stimulus.^1−4^ This interesting class of macromolecules has attracted a huge amount of attention within the literature over the past few decades, resulting in numerous examples of polymers and self-assembled nanostructures that respond to a variety of stimuli, including temperature,^5−9^ pH,^10−12^ light,^13−16^ oxidants/reductants,^17−19^ or enzymes.^20−22^ These external stimuli typically induce detectable micro- or nanoscale changes, which often result in significant variations in the macroscopic properties of the polymer, such as its shape, solubility, or mechanical properties.^1−4^ Due to the vast array of external stimuli that can induce a response, stimuli-responsive materials have come to be utilized in numerous applications, including drug delivery,^23,24^ interactive coatings,^25,26^ tissue engineering,^27,28^ and protein purification.^29,30^

While a wide range of polymers and nanostructures that respond to external stimuli have been reported to date, perhaps the most widely studied and best understood examples typically entail thermoresponsive polymers.^1−4^ In this case, thermoresponsive polymers will undergo a reversible change in solubility at a specific temperature, known as critical solution temperature (CST). Often, this transition temperature is also referred to as the cloud-point temperature (*T*_CP_).^31−33^ This is broadly used to classify thermoresponsive polymers into one of two categories, depending on whether they exhibit a lower-critical solution temperature (LCST) or an upper-critical solution temperature (UCST).^34−37^ LCST behavior corresponds to demixing of the polymer from solution above a critical temperature, whereas UCST behavior corresponds to an improved miscibility of the polymer with the solvent above a critical temperature.^34−37^ To date, a vast majority of literature examples utilizing temperature-sensitive polymers have focused on LCST-type phase transitions in aqueous media, with this disparity most commonly justified on the grounds that UCST behavior is rather sensitive to even small variations in pH, ionic strength, and (co)polymer composition, including end-group functionality.^34,35,38,39^

This interest in the use of LCST-type thermoresponsive polymers in aqueous solution can further be justified on the grounds that they can be regarded as simplified mimics of biological systems, which has driven studies into potential biomedical applications.^37,40,41^ For instance, one of the most extensively studied thermoresponsive polymers, poly(*N*-isopropylacrylamide) (PNIPAAm), and PNIPAAm-based assemblies have been widely explored in a range of biomedical applications such as drug carriers, enzyme mimics, and biosensors.^42−44^ However, the LCST of PNIPAAm is commonly reported to be approximately 32 °C, which can be a potential issue as it is below that of physiological temperature.^45,46^ Therefore, there is substantial research interest in being able to accurately tune the LCST of thermoresponsive polymers, in an effort to better control their thermoresponsive behavior and broaden their potential range of applications.

As such, a number of reports have demonstrated the ability to modulate the thermoresponsive behavior of LCST-type systems through various approaches, including the addition of chemical additives, such as salts^47^ and surfactants,^48^ upon varying the solution pH,^49^ or modifying the polymer composition, through the introduction of hydrophilic or hydrophobic functional groups or varying molecular weight, among other factors.^50−52^ For instance, Son et al. have provided evidence regarding the tunability of the LCST of linear poly(2-(dimethylamino)ethyl methacrylate) (PDMAEMA) by copolymerization with poly(*N,N′*-dimethyl(methacryloylethyl)ammonium propanesulfonate) (PDMAPS).^53^ More recently, Vamvakaki and co-workers reported the accurate modification of *T*_CP_ associated with the LCST of linear PDMAEMA by postpolymerization quaternization of the pendant tertiary amine groups.^54^ The amine groups were functionalized with halides to introduce functionalities of variable carbon chain lengths, targeting different degrees of quaternization. Overall, the *T*_CP_ of the quaternized PDMAEMA increased with increasing degree of quaternization, while increasing the length of the aliphatic chain of the halide resulted in reducing the *T*_CP_.^54^ Thus far, a limited number of studies have focused on the introduction of sulfobetaine functionalities within the structure of LCST-type polymers which possess pendant tertiary amine groups in order to modify their thermoresponsive behavior.^55,56^ Sulfobetaine-containing monomers possess zwitterionic character and contain a quaternary ammonium and a sulfonate group separated by a carbon spacer of variable chain length.^57,58^ While the quaternary ammonium and sulfonate groups are positively and negatively charged, respectively, the overall net charge of the structure is zero.^57,58^

Herein, we demonstrate a simple, yet efficient, method for regulating the thermoresponsiveness of cross-linked poly(2-(diethylamino)ethyl methacrylate) (PDEAEMA)-based block copolymer nanoparticles, prepared via RAFT-mediated emulsion polymerization, using a poly(*N,N′*-dimethyl(methacryloylethyl)ammonium propanesulfonate) (PDMAPS) steric stabilizer block, through a postpolymerization betainization approach. The original PDEAEMA-based nanoparticle platform was found to exhibit interesting thermoresponsive behavior, displaying an LCST in aqueous milieu, which eventually led to flocculation and macroscopic precipitation with increasing solution temperature in a reversible manner. Postpolymerization betainization of these nanoparticles using four different sulfonate salts was subsequently found to allow for modification of both *T*_CP_ and *T*_CFT_ depending on the type and molar ratio of the betainization reagent used. The thermoresponsive behavior of the resulting betainized particles was further explored with both *T*_CP_ and *T*_CFT_ and was found to increase with an increasing degree of betainization (i.e., increasing core hydrophilicity) in all cases. The identified relationships among the particle thermoresponsiveness, degree of functionalization, and sulfobetaine structure were also correlated to theoretical hydrophobicity calculations of oligomeric models possessing different degrees of betainization that resembled experimental conditions. Overall, this approach demonstrates a facile and versatile strategy for the preparation of thermoresponsive polymeric nanoparticles with tunable LCST and reversible aggregation behavior and should inform particle design in future studies with potential applications in drug delivery and biomimicry.

## Results and Discussion

**Scheme 1:**
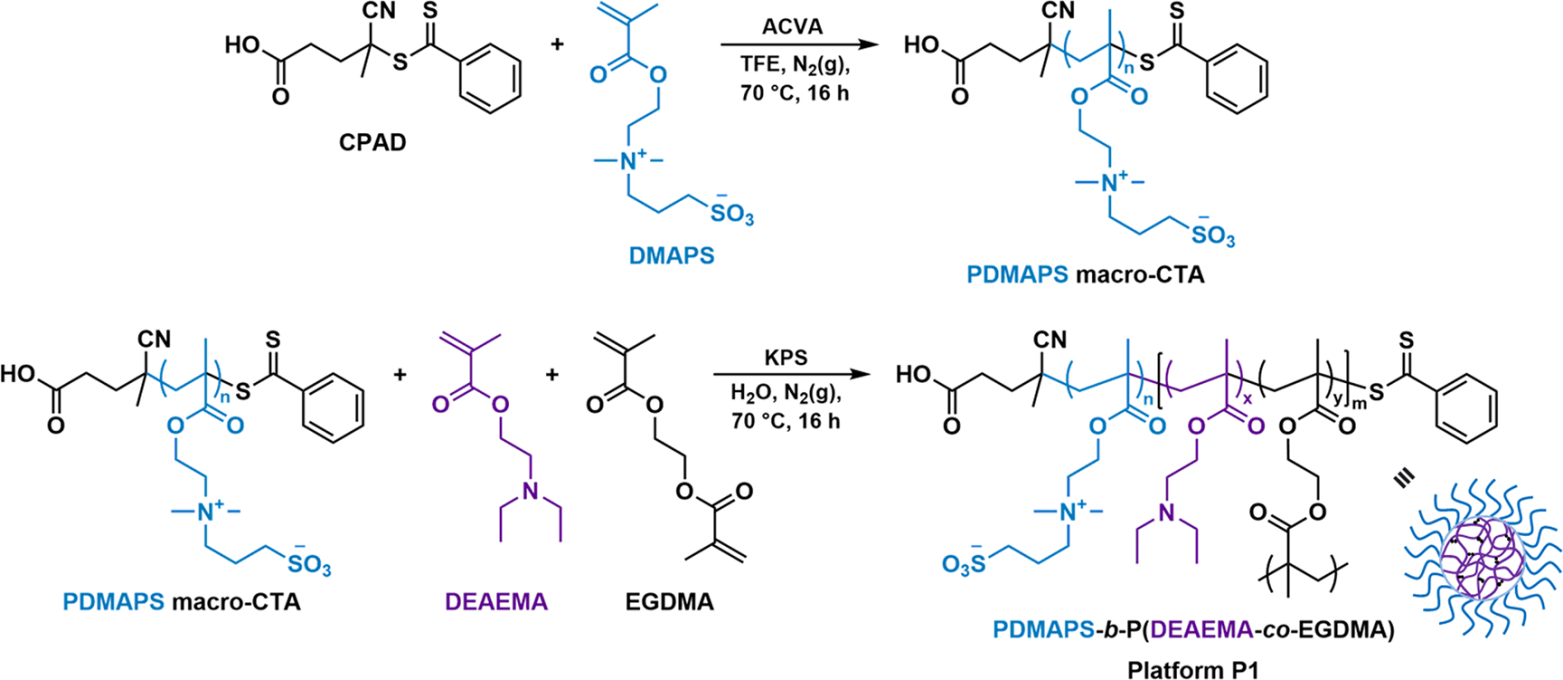
Reaction Scheme of the Synthetic Route Followed for the Preparation of PDMAPS_18_ Macro-CTA via RAFT Polymerization, and Subsequent Chain-Extension of DEAEMA and EGDMA via RAFT-Mediated Emulsion Polymerization to Form the Cross-Linked PDMAPS_18_-*b*-P(DEAEMA_675_-*co*-EGDMA_6_) **P1** Nanoparticles

We started our investigation by first preparing a water-miscible macromolecular chain-transfer agent (macro-CTA) via reversible addition–fragmentation chain-transfer (RAFT) polymerization. In this case, 4-cyano-4-(phenylcarbonothioylthio)pentanoic acid (CPAD) was utilized as the chain-transfer agent (CTA) for the homopolymerization of *N*,*N*′-dimethyl(methacryloylethyl)ammonium propanesulfonate (DMAPS), targeting a number-average molecular weight (*M*_n_) of 5000 Da. The polymerization was carried out at 70 °C for 16 h in 2,2,2-trifluoroethanol (TFE), using 4,4′-azobis(4-cyanovaleric acid) (ACVA) as the radical initiator (Scheme 1). After this period, monomer conversion of ∼90% was achieved, as confirmed by ^1^H NMR spectroscopy. Following purification by dialysis and lyophilization, the polymerization process to form the PDMAPS_18_ macro-CTA was found to be well-controlled, as indicated by ^1^H NMR spectroscopy and size-exclusion chromatography (SEC) (*M*_n,NMR_ = 5200 Da, *M*_n,SEC_ = 5600 Da, *Đ* = 1.11) (Figures S1 and S2, and Table S1). Furthermore, complete overlap between the recorded refractive index (RI) and UV (λ = 309 nm) traces in the SEC chromatogram confirmed the retention of the dithiobenzoate end-group of the macro-CTA and its suitability for further chain-extensions. The obtained PDMAPS_18_ macro-CTA was then utilized as the steric stabilizer in the oil-in-water RAFT-mediated emulsion copolymerization of 2-(diethylamino)ethyl methacrylate (DEAEMA) and ethylene glycol dimethacrylate (EGDMA) (Scheme 1). Polymerization was performed at 70 °C for 16 h in H_2_O, using potassium persulfate (KPS) as the radical initiator. This yielded the in situ formation of the targeted cross-linked PDMAPS_18_-*b*-P(DEAEMA_675_-*co*-EGDMA_6_) platform nanoparticles (**P1**) as a turbid aqueous dispersion at 5.25 wt % solids content.

**Figure 1:**
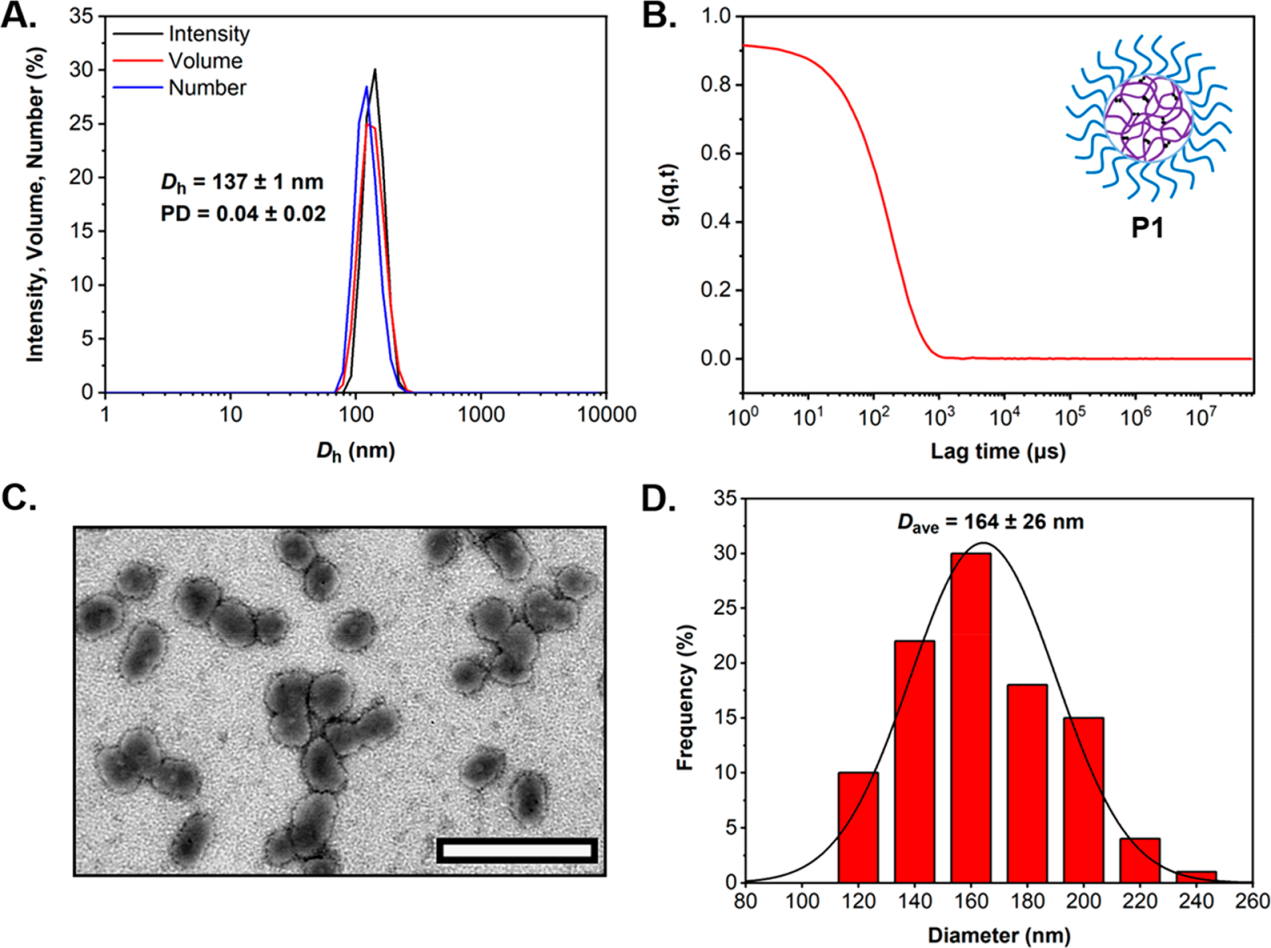
Characterization of cross-linked PDMAPS_18_-*b*-P(DEAEMA_675_-*co*-EGDMA_6_) **P1** particles. (A) DLS analysis showing the intensity, volume, and number-weighted size distributions along with average *D*_h_ and PD values (the error shows the standard deviation from 4 repeat measurements); (B) autocorrelation function as obtained by DLS at 15 °C, 0.1 mg mL^–1^ in 0.3 M NaCl solution at pH = 8.0; (C) representative dry-state TEM image, stained with 1 wt % uranyl acetate (UA) solution (scale bar represents 500 nm); and (D) histogram of particle size distribution along with calculated average diameter (*D*_ave_), measured from particle counting analysis of at least 100 particles based on the acquired TEM images.

Characterization of the resulting aqueous particle formulation by dynamic light scattering (DLS) and dry-state transmission electron microscopy (TEM) revealed the successful formation of a uniform population of spherical PDEAEMA-based core–shell nanoparticles (Figure 1 and Figure S3). DLS analysis at pH = 8.0 showed that the nanoparticles possessed an average hydrodynamic radius (*D*_h_) of 137 ± 1 nm with a corresponding polydispersity (PD) of 0.04 ± 0.02, displaying good overlap between the relative intensity, volume, and number size distributions (Figure 1A). Autocorrelation function obtained by DLS confirmed the uniformity of the particle formulation, whereby a smooth exponential decay and optimum signal-to-noise ratio were observed (Figure 1B). This data is in good agreement with the acquired representative dry-state TEM images, which revealed the formation of spherical nano-objects of uniform size (Figure 1C and Figure S3). Image analysis on the TEM data was subsequently performed to produce the corresponding histogram of particle size distribution, which suggested an average diameter (*D*_ave_) of 164 ± 26 nm for the PDEAEMA-based **P1** nanoparticles (Figure 1D). Overall, particles appear to be more irregular by TEM in comparison with results obtained by DLS analysis where the associated PD was rather low. It was suggested that the less homogeneous morphology observed by TEM could be a consequence of the dehydration of particles during the sample preparation. Further investigation by atomic force microscopy (AFM) confirmed that the formulation consisted of spherical nano-objects of uniform size and shape (Figure S3).

**Figure 2:**
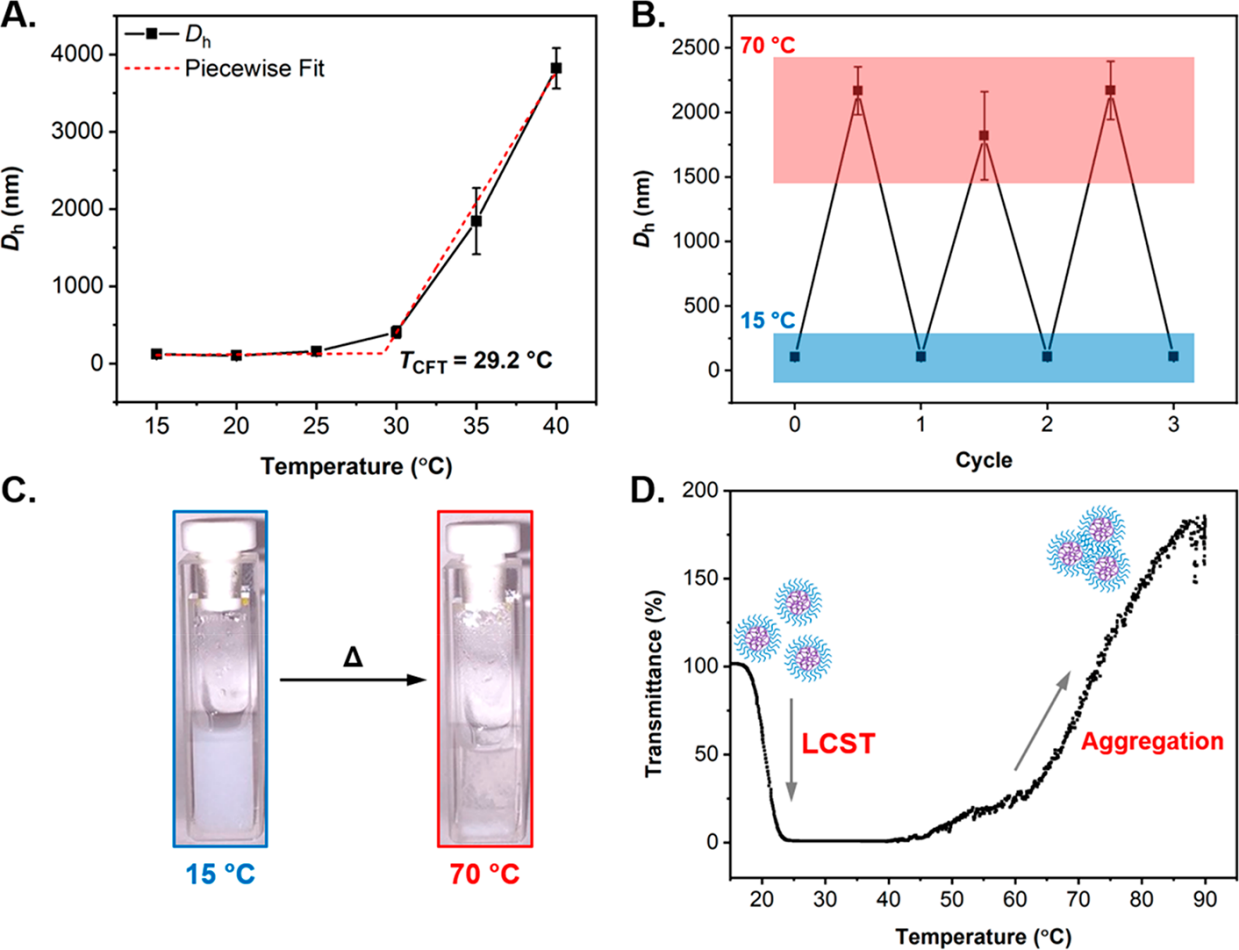
Evaluation of the thermoresponsive behavior of cross-linked PDEAEMA-based **P1** nanoparticles. (A) Variation in hydrodynamic radius (*D*_h_) with increasing solution temperature as measured by variable temperature DLS along with estimated *T*_CFT_, (B) reversible aggregation behavior for **P1** particles reporting changes in *D*_h_ as a function of solution temperature (data was recorded over 3 heating–cooling cycles from 15–70 °C in a single step of 55 °C by variable temperature DLS analysis), (C) images of the UV–vis cuvettes containing a 1 mg mL^–1^ solution of **P1** particles in 0.3 M NaCl at 15 °C (left) and after heating at 70 °C for 24 h (right), and (D) UV–vis transmittance spectra recorded at λ = 550 nm from 15 to 90 °C demonstrating the distinct LCST and aggregation behavior observed. All analysis was performed at a particle concentration of 1 mg mL^–1^ in 0.3 M NaCl solution at pH = 8.0.

The thermoresponsive behavior of the PDEAEMA-based **P1** platform nanoparticles was then investigated. Variable temperature DLS analysis, recorded at 1 mg mL^–1^ particle concentration, revealed that **P1** nanoparticles both increased in size and aggregated into larger clusters with increasing solution temperature (Figure 2A and Figure S14). The solution temperature was increased in 5 °C increments and was allowed to equilibrate for 5 min prior to analysis, revealing a distinct transition in *D*_h_ above 25 °C whereby the particles began to aggregate, with a maximum aggregate size >2 μm recorded at 40 °C. It was hypothesized that the observed increase in particle size was a consequence of a thermal transition resulting in a gradual destabilization of the particles, which in turn led to their macroscopic aggregation, a transition which is more commonly referred to as a critical flocculation temperature (*T*_CFT_).^59^ Similar aggregation behavior has been widely reported for LCST-type polymers.^36,60,61^ Therefore, it was hypothesized that the PDEAEMA-based cores undergo an LCST phase transition upon temperature increase.^62^ In this case, when heated above the trigger temperature of PDEAEMA, the particle cores became progressively more hydrophobic, which in turn reduced the stability of the particles. Then, in an effort by the system to reduce unfavorable interactions between PDEAEMA and water molecules, particles aggregated and precipitated from solution. While this could be something expected for LCST-type polymers, it should also be noted that particles were not observed to shrink prior to their aggregation upon an increase in the solution temperature. Given that the polymeric system studied in this work is core-cross-linked, the structure is relatively constrained, which therefore results in a reduction of the size variation associated with a thermal phase transition, such as an LCST. Furthermore, DLS measures the hydrodynamic diameter of the particles, and whereas their cores adopt a collapsed state above a critical temperature, their PDMAPS-rich shell remains solvated, which could prevent monitoring of such core size variation, especially if, as hypothesized, this variation is minor.

Using the Piecewise fitting tool, available in the OriginLab graphing software, a two-segment linear fit of the data was applied, identifying a *T*_CFT_ of 29.2 °C for the original PDEAEMA-based **P1** particles. Intrigued by this result, we decided to investigate it further. Using a single-step method for variable temperature DLS, where the particle size is first recorded at 15 °C, the solution is then rapidly heated to 70 °C, and the particle size is recorded again following equilibration for 5 min. We found that this thermoresponsive behavior was completely reversible, with no obvious change in particle *D*_h_ or PD observed at 15 °C over three heating–cooling cycles, providing that the aggregates were redispersed by agitation upon cooling (Figure 2B and Figure S3). The flocculation behavior was also observed macroscopically by simply leaving a 1 mg mL^–1^ aqueous solution of **P1** nanoparticles in an oven heated at 70 °C, where after 24 h of incubation time the particles had clearly sedimented at the bottom of the cuvette (Figure 2C). Finally, the observed thermoresponsive behavior of PDEAEMA-based platform nanoparticles was studied by variable temperature UV–vis spectroscopy, recording the solution transmittance between 15 and 90 °C at a fixed wavelength of λ = 550 nm. Interestingly, a sharp decrease in transmittance was observed below 25 °C followed by a steady increase in transmittance above 40 °C (Figure 2D). These findings confirmed that this phenomenon was a result of an LCST-type phase transition occurring below 25 °C with a *T*_CP_ = 20.5 °C, as the observed sharp decrease in transmittance is typical of LCST behavior recorded by UV–vis spectroscopy.^36^ This would then lead to destabilization of the particles triggering the flocculation event observed by UV–vis spectroscopy and DLS above 40 °C (Figure 2A,D). It was suggested that the temperature difference observed between the cloud-point and aggregation temperature was a consequence of the stabilizing effect provided by the PDMAPS corona-forming blocks. Indeed, to further increase the hydrophilicity of the particles, the antipolyelectrolyte effect of PDMAPS sulfobetaine units enhanced the stability of particles in saline media.^63,64^ As such, this resulted in increased particle stability above the cloud-point temperature, which could have delayed the occurrence of aggregation phenomena. In addition to the LCST phase transition, particles could theoretically have displayed a UCST associated with the PDMAPS-based shell. However, the molecular weight of corona-forming PDMAPS chains (ca. 5 kDa) is adequately short for them not to exhibit a UCST in the range of temperatures studied herein.^65^

While PDEAEMA is most commonly reported for its pH-responsiveness, there are also a limited number of reports regarding its temperature-responsive behavior.^62,66^ In comparison to the analogous poly(2-(dimethylamino)ethyl methacrylate) (PDMAEMA), which is often utilized as an LCST-type polymer,^62^ the thermoresponsive behavior of PDEAEMA in aqueous media has been underexplored due to its increased hydrophobicity and significantly lower solubility in water, contrary to PDMAEMA which is water-soluble across a broad pH range. Consequently, the LCST of PDEAEMA is typically observed within a narrower pH window (soluble < pH = 5 and insoluble > pH = 8).^62,66^ In this study, the thermoresponsive behavior of PDEAEMA-based particles was only observed within a much narrower pH window between pH = 7.8 and pH = 8.0. Above pH = 8.0, the PDEAEMA-based particle core becomes too hydrophobic to interact with water and display a phase transition. Conversely, reducing the pH progressively resulted in protonation of the tertiary amine groups located within the particle cores and subsequent enhancement of their hydrophilicity, which in turn suppressed their thermoresponsiveness. Consequently, all solution characterizations of PDEAEMA-based particles were performed at pH = 8.0.

**Scheme 2:**
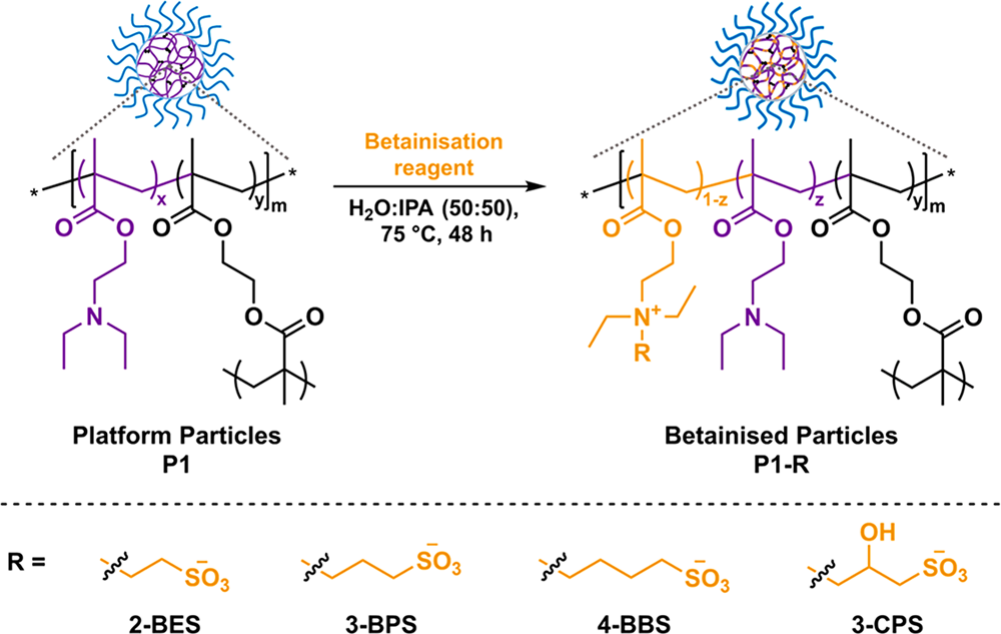
Reaction Scheme for the Postpolymerization Betainization Procedure Performed on the Cross-Linked PDEAEMA-Based **P1** Particles, Using Either 2-BES, 3-BPS, 4-BBS, or 3-CPS as the Betainization Reagent

Aiming to produce a versatile system which could attract interest in various fields of application, our efforts then turned to regulating the thermoresponsiveness of the originally prepared **P1** platform nanoparticles. The thermoresponsiveness modification was experimentally investigated upon varying the overall hydrophilicity/hydrophobicity of **P1** nanoparticles through a postpolymerization functionalization process in order to introduce sulfobetaine moieties within the particle cores. In this case, tertiary amine side groups of PDEAEMA repeating units were used as functional handles and converted into sulfobetaines following their reaction with sulfonate salt derivatives based on reported procedures for the preparation of hydroxysulfobetaines.^67^ The effect of both the sulfobetaine structure and the degree of functionalization over the cloud-point and flocculation temperature of resulting betainized particles was then explored. In this regard, four different betainization reagents, namely, sodium 2-bromoethanesulfonate (2-BES), 3-bromopropane sulfonate (3-BPS), sodium 4-bromobutane sulfonate (4-BBS), and sodium 3-chloro-2-hydroxy-1-propane sulfonate (3-CPS), were utilized in order to explore the effect of the sulfobetaine carbon chain length and the incorporation of hydroxyl functionalities alongside the modifier on the thermoresponsive behavior of the resulting particles. Betainization of the cross-linked **P1** particles using the aforementioned reagents was performed in a 1:1 mixture of H_2_O/isopropyl alcohol (IPA) at 75 °C for 48 h to ensure full reaction conversion, targeting degrees of betainization of 10%, 30%, 50%, and 100% relative to the number of PDEAEMA units (Scheme 2). Following betainization, the nanoparticles were purified by extensive dialysis, with the final dialysis medium change being performed in a 0.3 M NaCl aqueous solution (pH = 8.0). Each betainized particle dispersion was then diluted down to 5000 ppm solids content using 0.3 M NaCl(aq), yielding the targeted betainized **P1-R** nanoparticles (where **R** = 2-BES, 3-BPS, 4-BBS, or 3-CPS).

**Figure 3:**
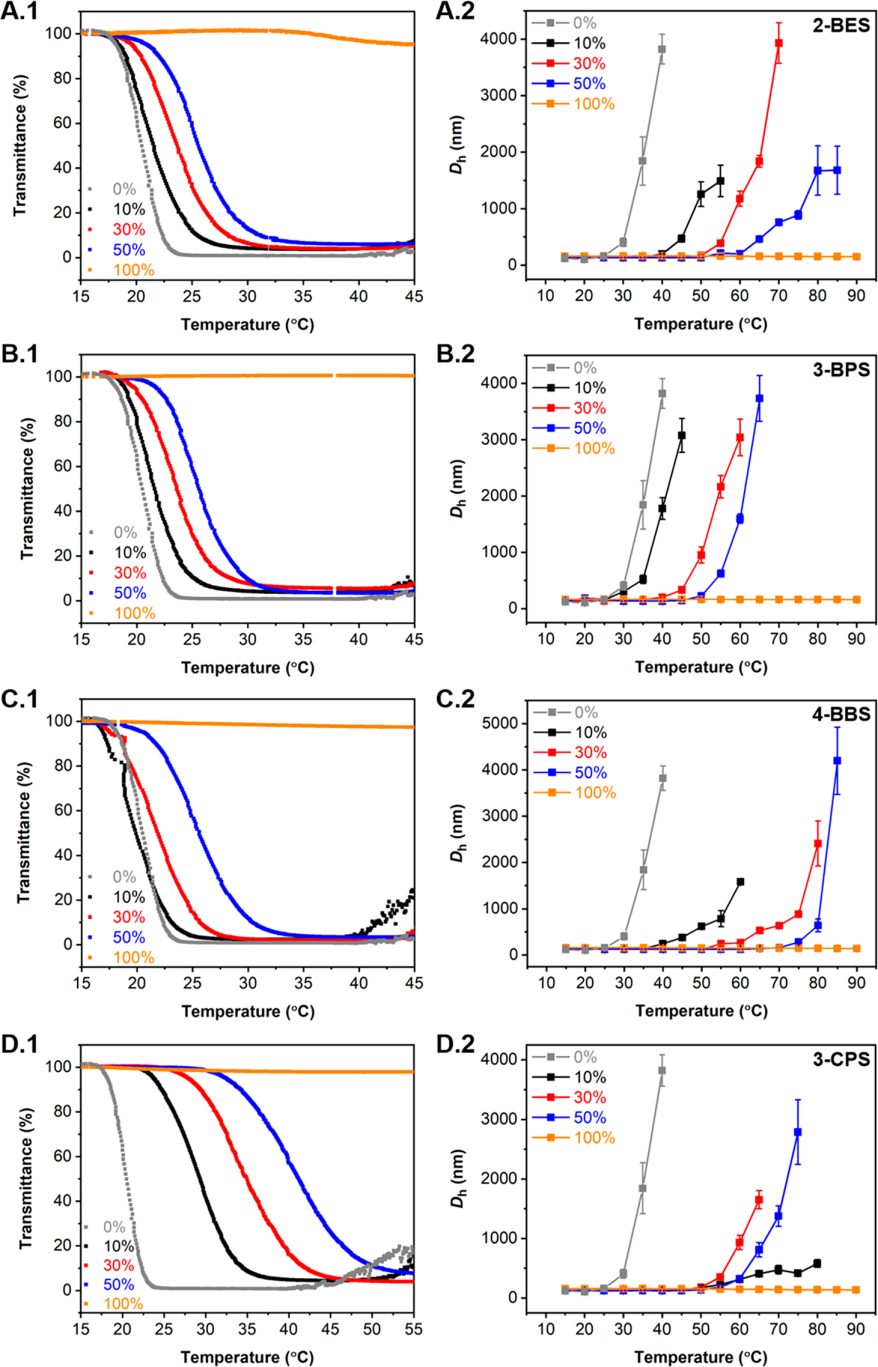
Evaluation of the thermoresponsive behavior of PDEAEMA-based nanoparticles betainized with (A) 2-BES (**P1-2-BES**), (B) 3-BPS (**P1-3-BPS**), (C) 4-BBS (**P1-4-BBS**), and (D) 3-CPS (**P1-3-CPS**) by (1) UV–vis spectroscopy and (2) variable temperature DLS analysis. Samples were run from 15 to 90 °C at 1 mg mL^–1^ in 0.3 M NaCl solution at pH = 8.0, with the UV–vis transmittance spectra recorded at λ = 550 nm used to determine *T*_CP_ and the DLS data used to determine *T*_CFT_, respectively.

Having prepared a library of betainized nanoparticles based on the **P1** platform upon varying both the degree of betainization and the betainization reagent used, we set about characterizing the resulting **P1-R** particles (**R** = 2-BES, 3-BPS, 4-BBS, or 3-CPS) and investigating their temperature-responsive nature by both DLS analysis and UV–vis spectroscopy (Figure 3 and Figures S4–S7, and Table 1). This was achieved using the same methods reported within Figure 2, using a 1 mg mL^–1^ solution of betainized particles in 0.3 M NaCl aqueous solution at pH = 8.0. Initial DLS analysis at 15 °C revealed that all betainized formulations possessed monomodal size distributions with *D*_h_ values that ranged between 115 and 159 nm and PD values between 0.03 and 0.07, whereas the spherical shape and uniformity of the nanoassemblies was verified by dry-state TEM and AFM imaging of the particles with 30% degree of betainization (**P1-R-30**) (Figures S4–S9). Interestingly, some key trends were observed in both the variable temperature DLS and UV–vis data, which appear to show an evident influence of the betainization reagent and degree of betainization on the thermoresponsive properties of the particles. Moreover, when going to extreme degrees of betainization, i.e., 100% relative to the number of PDEAEMA units within the particle cores, the temperature-responsive nature of the particles was completely suppressed. This is presumably attributed to the high degree of betainization within the particle cores and the subsequent marked increase in particle hydrophilicity, both of which would inhibit the LCST-triggered behavior of the nanoparticles. This is also evidenced by the noticeable increase in average particle size reported in Table 1 for **P1-3-BPS-100**, **P1-4-BBS-100**, and **P1-3-CPS-100** relative to those particles possessing 10%, 30%, and 50% degree of betainization.

**Table 1:** Summary of Nanoparticle *D*_h_, PD, *T*_CP_, and *T*_CFT_ Data Following Betainization of PDEAEMA-Based **P1** Nanoparticles Using 10, 30, 50, and 100 mol % of 2-BES (**P1-2-BES**), 3-BPS (**P1-3-BPS**), 4-BBS (**P1-4-BBS**), or 3-CPS (**P1-3-CPS**)

Sample	% Betainization	*D*_h_ (nm)	PD	*T*_CP_ (°C)	*T*_CFT_ (°C)
**P1**		137	0.04	20.5	29.2
**P1-2-BES-10**	10	135	0.04	21.6	42.1
**P1-2-BES-30**	30	137	0.06	23.6	53.5
**P1-2-BES-50**	50	126	0.07	25.6	58.7
**P1-2-BES-100**	100	128	0.04		
**P1-3-BPS-10**	10	115	0.06	21.6	33.6
**P1-3-BPS-30**	30	127	0.06	23.6	43.9
**P1-3-BPS-50**	50	133	0.04	25.6	56.6
**P1-3-BPS-100**	100	159	0.03		
**P1-4-BBS-10**	10	132	0.03	19.9	39.3
**P1-4-BBS-30**	30	121	0.04	21.9	56.3
**P1-4-BBS-50**	50	119	0.07	25.6	79.3
**P1-4-BBS-100**	100	156	0.05		
**P1-3-CPS-10**	10	128	0.06	29.3	47.0
**P1-3-CPS-30**	30	125	0.04	35.0	53.3
**P1-3-CPS-50**	50	125	0.04	41.2	60.4
**P1-3-CPS-100**	100	149	0.07		

Considering the *T*_CP_ values recorded by UV–vis spectroscopy, relatively similar trends were observed for **P1** particles functionalized with varying degrees of either 2-BES (**P1-2-BES-10**, **-30**, **-50**), 3-BPS (**P1-3-BPS-10**, **-30**, **-50**), or 4-BBS (**P1-4-BBS-10**, **-30**, **-50**), which indicated that changing the carbon length of the sulfobetaine modifier had no evident effect on the resulting *T*_CP_. Furthermore, it was observed that *T*_CP_ increased only by 5 °C with an increasing degree of incorporation of these aliphatic sulfobetaine functionalities from 10% to 50% irrespective of the structure of the betainization reagent (Figure 3A.1–C.1 and Table 1). In contrast to aliphatic sulfonate salts, betainization of **P1** platform particles with 3-CPS (**P1-3-CPS-10**, **-30**, **-50**) demonstrated a much more drastic effect on the resulting *T*_CP_ over a broader temperature range, which was shown to increase by up to 20.7 °C for **P1-3-CPS-50** (Figure 3D.1 and Table 1). It was therefore suggested that this marked change of *T*_CP_ when using 3-CPS resulted from the enhanced hydrophilicity within the particle core introduced by the hydroxyl moieties alongside the carbon spacer of the sulfobetaine, which increased the level of particle hydration by solvent molecules. On the basis of variable temperature DLS analysis, a correlation between the *T*_CFT_ and the degree of betainization was retained, with a clear impact of the degree of betainization on the response temperature being observed regardless of the sulfobetaine structure (Figure 3A.2–C.2 and Figures S15–S26, and Table 1). However, our findings indicated that *T*_CFT_ could not be directly correlated to the structure of the sulfobetaine utilized in each case. Indeed, it was quite unexpected to observe the formation of aggregates at higher temperature for **P1-4-BBS-50** in comparison to **P1-3-CPS-50**, whereby particles betainized using 4-BBS should be less hydrophilic than those modified with 3-CPS at identical degrees of functionalization. Furthermore, although particle flocculation was typically observed to occur at higher temperatures than LCST, the much broader range of temperature response observed for *T*_CFT_ could indicate that the betainization had a greater repercussion on the flocculation temperature, especially for particles being functionalized with 4-BBS. In the case of **P1-3-CPS** particles, flocculation was displayed over a narrower temperature range. They did however present a much higher initial *T*_CFT_ of 47.0 °C at only 10% degree of betainization, verifying that increased core solvophilicity is indeed a key parameter in determining the thermoresponsive behavior of self-assembled nanostructures. Similar to PDEAEMA-based platform particles, betainized particles were not observed to shrink by DLS prior to their aggregation, as a result of the minor size variation of the cross-linked cores when heated above the LCST of PDEAEMA. Overall, variable temperature DLS analysis indicated a clear impact of the betainization process on the *T*_CFT_, even when varying the aliphatic chain of the sulfobetaine, whereas it only showed a minor variation over the *T*_CP_ that was more pronounced for the most hydrophilic betainization reagent. Moreover, it was speculated that the temperature difference observed between *T*_CP_ and *T*_CFT_ suggests that the aggregation of particles occurs at a higher temperature as a consequence of their LCST behavior and therefore that particle responsiveness transits via a metastable state between the LCST and the onset of aggregation, which stems from the enhanced stability induced by the PDMAPS shell and is more susceptible to variability. Furthermore, the formed particle aggregates are expected to produce stronger scattering in DLS, which will directly result in a sharp increase of the signal and measured *D*_h_ values even if this is not representative of the whole sample. Consequently, DLS findings are potentially less representative of the whole body of each sample, while transmittance measurements by UV–vis analysis are more illustrative and reliable toward determining the LCST phase transition for each formulation. Importantly, no UCST phase transition was observed following betainization of PDEAEMA units, even when the particle core was completely betainized. Again, particle characterization was performed in 0.3 M NaCl solution, which was considered to suppress the UCST of poly(sulfobetaine)s through charge screening.^34^ In addition, the cross-linked core constrained most of the sulfobetaine moieties, and consequently, interparticle interactions were only possible through the PDMAPS-based corona. However, as discussed above for **P1** platform particles, the PDMAPS steric stabilizer block had a molecular weight of ca. 5 kDa, which is not expected to exhibit a UCST in the temperature range examined.^65^

Furthermore, the reversibility of the thermoresponsiveness of the betainized particles was assessed upon monitoring *D*_h_ and PD variations over multiple heating–cooling cycles by variable temperature DLS analysis for particles with 30% degree of betainization (**P1-R-30**), in a manner similar to that shown in Figure 2B for **P1**, indicating a completely reversible behavior and nanoparticles that retain their original size upon cooling to 15 °C (Figures S10–S13). On the basis of these findings, it seems apparent that the use of a postpolymerization betainization approach can be utilized as an efficient method to facilitate the tuning of the *T*_CP_ values in thermoresponsive polymeric nanoparticles. These findings also indicated a clear correlation between the degree of betainization and the *T*_CFT_, while no apparent trend was identified between the measured *T*_CFT_ and the structure of the sulfobetaine used.

**Figure 4:**
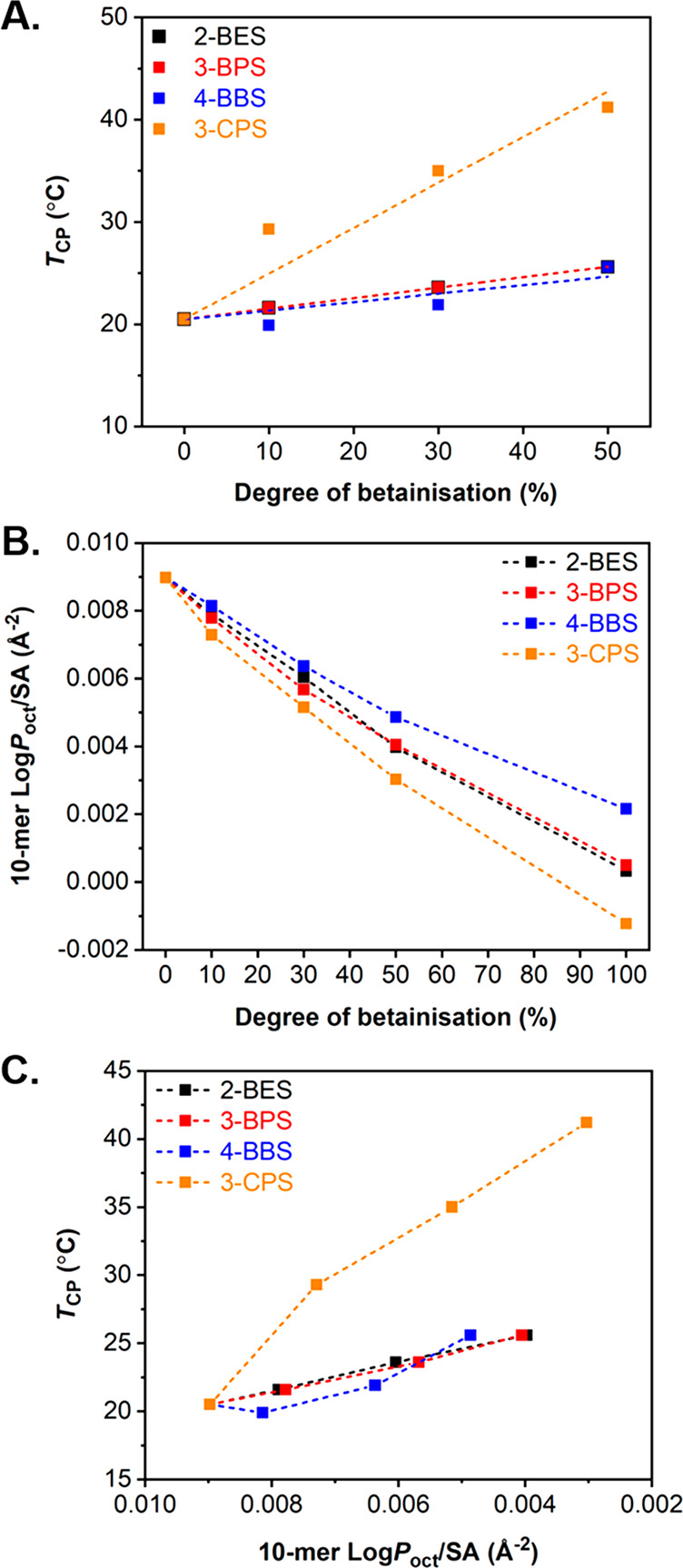
(A) Correlation plot for measured *T*_CP_ values as a function of the degree of betainization for each betainization reagent used, (B) evolution of 10-mer hydrophobicity as a function of the degree of betainization for each betainization reagent used, and (C) correlation of *T*_CP_ as a function of increasing 10-mer hydrophilicity (i.e., decreasing Log *P*_oct_/SA values). Log *P*_oct_ values (*A* Log *P* method) were calculated using an atom-based approach and normalized by solvent-accessible surface area (SA) using Materials Studio 2020.

It was then aimed to further investigate the correlation between degree of betainization and *T*_CP_ in more detail. For this study, a linear fitting for *T*_CP_ values as a function of the degree of betainization was performed using OriginLab software for each betainization reagent (Figure 4A). The linear trend was passed through a fixed point corresponding to the *T*_CP_ of the original PDEAEMA-based **P1** nanoparticles to determine the extent of the effect the postpolymerization approach has on the thermoresponsive behavior of the resulting betainized particles. The linear fit can then be used to calculate both the slope and the Pearson’s correlation coefficient (PCC) of this data, providing an indication of the linear correlation between *T*_CP_ and the degree of betainization (Table S2). While a clear effect of the postpolymerization betainization process was observed on *T*_CFT_, no fitting was performed in this case due to the difficulty to further elucidate the trend observed by DLS, which could be associated with instrumentation limitations to fully characterize this type of transition as discussed above (Figure S27). As was previously observed, the use of aliphatic sulfobetaines of different chain lengths appears to have a minimal impact on the *T*_CP_, with slopes equal to or less than 0.1 recorded for each one of 2-BES, 3-BPS, and 4-BBS reagents. In comparison, 3-CPS was found to be much more impactful, with a slope of 0.45 observed for the linear fit of this betainization reagent, which is likely due to the additional hydroxyl functionality and increased hydrophilicity introduced, facilitating increased solvation of the nanoparticle cores by solvent molecules that, in turn, leads to a concurrent increase of *T*_CP_. Regarding correlation, in all cases PCC values of greater than 0.81 were calculated, suggesting a strong, positive linear correlation in the data for the influence of degree of betainization on *T*_CP_ regardless of the structure of the sulfobetaine utilized.

Ultimately, we aimed to investigate the correlation between the *T*_CP_ measured by UV–vis spectroscopy with the core hydrophilicity of the corresponding particles upon betainization. The hydrophobicity (or hydrophilicity) of a molecule can be quantified by calculating its Log*P*_oct_ value, which describes the partitioning of a substance between an octanol-rich and water-rich environment, following a theoretical atom-based approach.^52^ In order to minimize the variability associated with polymer molecular weight and end-group discrepancies, the reported Log*P*_oct_ values were normalized by solvent-accessible surface area (SA).^51,52,68^ Log*P*_oct_/SA values can either be positive or negative depending on the preference of the polymer to partition in the octanol or the water phase, respectively.^52^ In our study, Log*P*_oct_/SA values were calculated for 10-meric DEAEMA-based models, resembling the core chemistry of **P1** particles, with degrees of betainization ranging from 10%, 30%, 50%, to 100% for all four betainization reagents utilized (Scheme S1). Calculated Log*P*_oct_/SA values correlated to the degree of betainization in each case displayed an apparent relationship between the oligomer hydrophobicity, the nature of the betainization reagent used, and the degree of betainization (Figure 4B). In particular, it was found that Log*P*_oct_/SA values decreased with an increasing degree of betainization in all cases, with 3-CPS having the most profound effect in increasing oligomer hydrophilicity. Furthermore, this theoretical investigation further supported our original hypothesis that the hydrophilicity within the nanoparticle cores increased with either increasing the sulfobetaine content or by using betainization reagents, such as 3-CPS, possessing hydrophilic moieties in their structure. While there is an overlap for Log*P*_oct_/SA values associated with DEAEMA-based 10-mers functionalized with 2-BES and 3-BPS, as well as a gap when using 3-CPS, which correlate with results reported by UV–vis spectroscopy, the higher Log*P*_oct_/SA values calculated for 4-BBS as compared to 2-BES and 3-BPS also indicated that the method can be somewhat limited for the accurate prediction of the core hydrophobicity. Furthermore, it should be noted that Log*P*_oct_/SA differences based on the nature of betainization reagent used were less evident at lower degrees of betainization (≤30%). An additional correlation of measured *T*_CP_ values for each betainization reagent with computationally calculated oligomer hydrophobicity values revealed a gradual *T*_CP_ increase with decreasing 10-mer Log*P*_oct_/SA (i.e., increasing hydrophilicity as the betainization degree increased), again with 3-CPS showing the biggest increase in *T*_CP_ at the same degree of betainization compared to the rest of the reagents used (Figure 4C). However, it was also evident that *T*_CP_ was only seen to significantly vary upon varying the degree of betainization of 3-CPS, while Log*P*_oct_/SA values trended with a relatively similar manner for every sulfobetaine examined. Therefore, at this stage, a theoretical hydrophobicity evaluation based on 10-meric oligomers could not directly be implemented to accurately predict *T*_CP_ of further particle formulations. Nonetheless, these results confirm that cloud-point temperature correlates to the core hydrophobicity of thermoresponsive particles, which is strongly associated with the degree of betainization and the structure of the sulfobetaine utilized in the case of 3-CPS, but to a lesser extent for aliphatic sulfobetaines of varying chain length.

## Conclusions

In summary, we have reported the synthesis of cross-linked PDEAEMA-based particles through RAFT-mediated emulsion polymerization of DEAEMA and EGDMA, using a PDMAPS steric stabilizer block. The resulting well-defined core–shell nanoparticles were found to exhibit reversible thermoresponsive properties, demonstrating both a *T*_CP_ and *T*_CFT_ with increasing solution temperature, a consequence of the PDEAEMA-based core possessing an LCST in aqueous media. Using the PDEAEMA units as functional handles, the core of the originally obtained particles was subsequently modified via a postpolymerization betainization approach employing a series of sulfonate salts of varying nature and hydrophilicity. Overall, it was demonstrated that both *T*_CP_ and *T*_CFT_ increased upon increasing the degree of betainization until the thermoresponsive behavior was lost at 100% degree of betainization, as a result of the cores being completely functionalized with sulfobetaine moieties. Regarding the structure of betainization reagent used, it was found that *T*_CP_ increased considerably only for particles being functionalized with 3-CPS, owing to the presence of hydroxyl functionalities which enhanced the hydrophilicity of the resulting particles, whereas aliphatic sulfobetaine functionalities of varying length (i.e., 2-BES, 3-BPS, and 4-BBS) had only a minor effect on the *T*_CP_. In addition, particle flocculation was observed to occur at higher temperatures and varied over a much wider temperature range when varying the degree of betainization, regardless of the sulfobetaine functionality utilized. However, no appreciable correlation between the sulfobetaine structure and the *T*_CFT_ could be obtained. Attempts to produce a model to correlate *T*_CP_ of betainized PDEAEMA-based particles and computationally calculated Log*P*_oct_/SA values of 10-meric DEAEMA-based oligomers with different degrees of betainization partially verified the experimentally observed findings and will require further development to be used as predictive tool in future studies. Despite the relative limitation of this approach to precisely tune the trigger temperatures of tertiary amine-based particles, the obtained results showcase our postpolymerization betainization approach as a simple and straightforward method for modifying the thermoresponsive behavior of polymeric particles in aqueous media with potential applications in drug delivery, catalysis, and biomimicry, among others.

## Methods

### Synthesis of Poly(*N*,*N*′-Dimethyl(methacryloylethyl)ammonium propanesulfonate) (PDMAPS) Macro-CTA

*N*,*N*′-Dimethyl(methacryloylethyl)ammonium propanesulfonate (DMAPS) monomer (5 g, 17.89 mmol, 18 equiv), 4-cyano-4-(phenylcarbonothioylthio)pentanoic acid (CPAD) chain-transfer agent (CTA) (0.28 g, 0.99 mmol, 1 equiv), and 4,4′-azobis(4-cyanovaleric acid) (ACVA) radical initiator (0.06 g, 0.20 mmol, 0.2 equiv) were dissolved in 2,2,2-trifluoroethanol (TFE) (25 mL). After transferring the solution to an ampoule equipped with a magnetic stir bar, the solution was degassed by purging with N_2_(g) for 30 min under rapid stirring. The polymerization reaction was initiated upon immersion of the ampoule in an oil bath heated at 70 °C, and the polymerization mixture was stirred at this temperature for 16 h to ensure full monomer conversion. The polymerization reaction was then terminated upon cooling and exposing the polymerization mixture to air. The resulting PDMAPS_18_ macro-CTA was purified by extensive dialysis against deionized water (MWCO = 1 kDa) and was recovered as a pink solid by lyophilization (3.65 g, 0.65 mmol). The resulting polymer was then characterized by ^1^H NMR spectroscopy and aqueous SEC analysis (Figures S1 and S2 and Table S1). ^1^H NMR (400 MHz, D_2_O + 0.5 M NaCl) conv ∼90%, *M*_n,NMR_ = 5200 g mol^–1^. SEC (H_2_O/MeOH (80:20) + 0.1 M NaNO_3_) *M*_n,SEC_ = 5600 g mol^–1^, *Đ*_SEC_ = 1.11.

### Synthesis of Cross-Linked PDMAPS-*b*-P(DEAEMA-*co*-EGDMA) Platform Particles (**P1**) via RAFT-Mediated Emulsion Polymerization Using PDMAPS_18_ Macro-CTA as Steric Stabilizer

PDMAPS_18_ macro-CTA (*M*_n,NMR_ = 5.2 kDa) (0.1 g, 0.02 mmol, 1 equiv), 2-(diethylamino)ethyl methacrylate (DEAEMA) monomer (2.5 g, 13.5 mmol, 675 equiv), and ethylene glycol dimethacrylate (EGDMA) cross-linking monomer (0.025 g, 0.13 mmol, 6.5 equiv) were dispersed in water (47 mL) having a resistivity of 18.2 MΩ cm by rapid stirring (in the order listed). After transferring the solution to an ampoule equipped with a magnetic stirrer bar, the resulting mixture was purged with N_2_(g) for 30 min under rapid stirring and then heated for 30 min in an oil bath heated at 70 °C. The radical initiator potassium persulfate (KPS) (0.025 g, 0.09 mmol, 4.5 equiv) was dissolved separately in water (1 mL) having a resistivity of 18.2 MΩ cm and purged with N_2_(g) for 10 min. The degassed KPS solution was added to the degassed macro-CTA/monomer solution to initiate polymerization. The resulting polymerization mixture was then stirred at 600 rpm at a temperature of 70 °C for 16 h to ensure full monomer conversion. The polymerization reaction was then terminated upon cooling and exposing the polymerization mixture to air. This procedure resulted in in situ emulsion polymerization-induced self-assembly (PISA), yielding the obtained PDEAEMA-based **P1** particles as an aqueous dispersion, which was further analyzed by DLS, UV–vis spectroscopy, and dry-state TEM and AFM imaging (Figures 1 and 2 and Figure S3).

### Synthesis of Sodium 4-Bromobutanesulfonate (4-BBS)

A suspension of anhydrous sodium bromide (1.13 g, 11 mmol, 1.1 equiv) and 1,4-butane sultone (1.36 g, 10 mmol, 1.0 equiv) in DMF (10 mL) was heated in an oil bath maintained at 80 °C for 2 h under constant stirring. The resulting clear solution was cooled to room temperature, and a white solid precipitated. The mixture was diluted with ethyl acetate and then filtered. The white solid obtained was washed five times with diethyl ether and dried in vacuo to give the target material sodium 4-bromobutanesulfonate (4-BBS) (2.18 g, 91% yield) as a white powder. ^1^H NMR (400 MHz, D_2_O): δ (ppm) 1.84 (m, 4H), 2.84 (t, 2H), 3.44 (t, 2H).

### Betainization of PDEAEMA-Based **P1** Particles Using Sodium 2-Bromoethanesulfonate (2-BES), Sodium 3-Bromopropanesulfonate (3-BPS), Sodium 4-Bromobutanesulfonate (4-BBS), or Sodium 3-Chloro-2-hydroxy-1-propanesulfonate (3-CPS)

Either sodium 2-bromoethanesulfonate (2-BES), sodium 3-bromopropanesulfonate (3-BPS), sodium 4-bromobutanesulfonate (4-BBS), or sodium 3-chloro-2-hydroxy-1-propanesulfonate (3-CPS) (molar equivalents based on PDEAEMA units in the precursor **P1** nanoparticles ×0.1, 0.3, 0.5, or 1 for targeting different degrees of betainization) and NaOH (20 mL of 0.2 M aqueous solution, 0.05 mol equiv based on PDEAEMA units in the precursor **P1** nanoparticles) were added portion-wise to a dispersion of **P1** particles in H_2_O:IPA (50:50, 300 mL) (concentration of precursor **P1** particles = 50 mg mL^–1^). The particle dispersion was heated at a temperature of 75 °C for 48 h under rapid stirring. Unreacted sulfonate, propan-2-ol cosolvent, and sodium salt byproducts were removed via extensive dialysis against deionized water (MWCO = 6–8 kDa) with the final cycle performed in 0.3 M NaCl at pH = 8.0. The resulting betainized **P1-R** nanoparticles were obtained as a dispersion in water, which was further analyzed by DLS, UV–vis spectroscopy, and dry-state TEM and AFM imaging.

## Supporting Information Available

Materials and characterization techniques, oligomer hydrophobicity evaluation details, supplementary NMR and SEC analysis data for PDMAPS macro-CTA, supplementary DLS analysis and TEM/AFM imaging data for PDMAPS-*b*-P(DEAEMA-*co*-EGDMA) (**P1**) and betainized **P1-R** particles, supplementary variable temperature DLS analysis data for thermoresponsive behavior assessment, and *T*_CFT_ determination of **P1-R** particles (PDF)
