# Supplementary material for: Tuning the Cloud-Point and Flocculation Temperature of Poly(2-(diethylamino)ethyl methacrylate)-Based Nanoparticles via a Postpolymerization Betainization Approach
Source: ACS Polym Au. 2021 Jul 8;1(1):47–58. doi: 10.1021/acspolymersau.1c00010 (PMC8389998; doi:10.1021/acspolymersau.1c00010)
Supplement: Supplementary file 1 — lg1c00010_si_001.pdf [file lg1c00010_si_001.pdf]

Supporting Information

Tuning the Cloud Point and Flocculation Temperature of Poly(2-(Diethylamino)ethyl Methacrylate)-Based Nanoparticles *via* a Post-Polymerization Betainisation Approach

Matthieu P. J. Miclotte,[1] *Stefan B. Lawrenson,*[1] *Spyridon Varlas,*[1] *Bilal Rashid,*[2] *Emma Chapman*[2] *and Rachel K. O'Reilly**[,1]

[1]School of Chemistry, University of Birmingham, Edgbaston, Birmingham, B15 2TT, UK

[2]BP Exploration Operating Company Ltd., Chertsey Road, Sunbury-on-Thames, Middlesex, TW16 7LN, UK

**Corresponding Author*: r.oreilly@bham.ac.uk (R.K.O.R.)

Contents

Materials S3

Characterization techniques S3

Evaluation of oligomer hydrophobicity S6

Characterization data for poly(*N*,*N*'-dimethyl(methacryloylethyl)ammonium propane sulfonate) (PDMAPS) macro-CTA.. S8

Supplementary characterization data for PDMAPS-*b*-P(DEAEMA-*co*-EGDMA) platform particles (**P1**) S10

DLS analysis of PDEAEMA-based **P1** particles betainised with 10, 30, 50 and 100 mol% 2-BES (**P1-2-BES-10, 30, 50, 100**) S11

DLS analysis of PDEAEMA-based **P1** particles betainised with 10, 30, 50 and 100 mol% 3-BPS (**P1-3-BPS-10, 30, 50, 100**) S12

DLS analysis of PDEAEMA-based **P1** particles betainised with 10, 30, 50 and 100 mol% 4-BBS (**P1-4-BBS-10, 30, 50, 100**) S13

DLS analysis of PDEAEMA-based **P1** particles betainised with 10, 30, 50 and 100 mol% 3-CPS (**P1-3-CPS-10, 30, 50, 100**) S14

Representative dry-state TEM and AFM images of **P1** particles betainised with (A) 2-BES, (B) 3-BPS, (C) 4-BBS, and (D) 3-CPS at 30 mol% (**P1-R-30**) S15

Reversibility of the thermoresponsive behavior for the betainised **P1-R** particles S17

T_{CFT} calculations for the betainised **P1-R** particles by Piecewise linear fitting S21

Materials

N,*N*'-Dimethyl(methacryloylethyl)ammonium propane sulfonate (DMAPS) (95%), 4-cyano-4-(phenylcarbonothioylthio)pentanoic acid (CPAD), potassium persulfate (KPS) (≥99.0%), sodium 2-bromoethanesulfonate (2-BES) (98%), sodium 3-chloro-2-hydroxy-1-propane sulfonate (3-CPS) (95%), 1,4-butane sultone (≥99%), 2,2,2-trifluoroethanol (TFE) (≥99%), and deuterium oxide (D_2O) were obtained from Sigma-Aldrich and were used as received. 2-(Diethylamino)ethyl methacrylate (DEAEMA) (99%) and ethylene glycol dimethacrylate (EGDMA) (98%) were also obtained from Sigma-Aldrich and were passed through a column of basic alumina for inhibitor removal prior to use. 4,4'-Azobis(4-cyanovaleric acid) (ACVA) (98%) and sodium 3-bromopropane sulfonate (3-BPS) (97%) were obtained from Alfa Aesar and were used as received. Sodium chloride (NaCl), sodium bromide (99+%, anhydrous) (NaBr), sodium hydroxide (NaOH) (98.5%), *N*,*N*-dimethylformamide (DMF) (99.5%), ethyl acetate (EtOAc) (99%) and isopropyl alcohol (IPA) (99.5%) were obtained from Fischer Scientific. Spectrum Spectra/Por dialysis tubing with either 1 kDa or 6–8 kDa MWCO was supplied by Fischer Scientific. Deionized and 18.2 MΩ·cm ultrapure water was obtained from a Triple Red Alto purification system and was used in all experiments unless stated otherwise.

Characterization techniques

NMR Spectroscopy. ^{1}H- and ^{13}C-Nuclear magnetic resonance (NMR) spectra were acquired using a Bruker DPX-400 spectrometer using deuterium oxide (D_2O) as the solvent at room temperature. Chemical shifts are reported as δ in parts per million (ppm) and are relative to solvent residual peaks (HDO, δ = 4.79 ppm).

Size Exclusion Chromatography. Molecular weight distributions were determined using aqueous size exclusion chromatography (SEC) on an Agilent PL50 instrument fitted with an Agilent PL aquagel-OH MIXED-M column (300 × 7.5 mm × 5 µm) and an aquagel guard column. The mobile phase used was H_2O:MeOH (80:20) containing 0.1 M $NaNO_3$, at a flow rate of 1.0 mL min^{-1}, a column temperature of 35 °C and detection by both refractive index (RI) and single wavelength UV at λ = 309 nm. 80 µL of sample were injected for each measurement and eluted for 35 min. Samples were prepared at a concentration of 1 mg mL^{-1} and filtered using a 0.45 µm nylon filter prior to analysis. Calibration was carried out in the molecular weight range 100-30000 Da using EasiVial polyethylene glycol (PEG) standards supplied by Agilent Technologies.

Dynamic Light Scattering. Dynamic light scattering (DLS) experiments were performed using either a Malvern Zetasizer Nano S or ZSP system equipped with a 633 nm He-Ne laser at either 4 mW or 10 mW, respectively. All size measurements were made using samples of concentration 0.1 mg mL^{-1} in 0.3 M $NaCl_{(aq)}$ (pH = 8.0) at 15 °C, with light scattering detected at an angle of 173° (back-scattering). Hydrodynamic diameters (D_h) were determined using the Stokes-Einstein equation, which assumes perfectly monodisperse non-interacting spheres, and averaged over 4 consecutive runs with at least 10 measurements recorded for each run. Variable temperature DLS analysis was performed from 15–90 °C in 5 °C intervals, with the sample equilibrated at the set temperature for 300s. Samples were prepared at a concentration of 1 mg mL^{-1} in 0.3 M $NaCl_{(aq)}$ (pH = 8.0).

UV-Vis Spectroscopy. UV-Vis spectroscopy was performed using a Thermo Scientific Evolution 350 UV-Vis spectrophotometer equipped with a Xenon flash lamp light source and a dual-matched silicon photodiode detector. Quartz cells (360–2500 nm) from Hellma with two polished sides were used for examining the transmittance spectral data by using Thermo INSIGHT-2

v.10.0.30319.1 software. A thermostat and 8-cell Peltier system with precise temperature control between 0 °C and 90 °C were coupled with Evolution 350 UV-Vis spectrophotometer to record T_{CP} values by recording temperature-dependent transmittance spectra of each sample from 15–90 °C.

Transmission Electron Microscopy. Dry-state stained transmission electron microscopy (TEM) imaging was performed on a JEOL JEM-1400 microscope at an acceleration voltage of 80 kV. All samples were diluted with deionized water to appropriate analysis concentration and then deposited onto formvar-coated copper grids. After approximately 1 min, excess sample was blotted from the grid and the grid was stained using an aqueous 1 wt% uranyl acetate (UA) solution for 1 min prior to blotting, drying and microscopic analysis. Average particle diameters (D_{ave}) were determined by measuring 100 particles per sample using the ImageJ software.

Atomic Force Microscopy. Atomic force microscopy (AFM) imaging was performed on a JPK NanoWizard4 microscope. Samples were drop cast from 1 mg mL^{-1} solutions in 0.3 M $NaCl_{(aq)}$ onto a silicon wafer. The substrate was dried under a gentle flow of compressed air prior to imaging. The tips for the AFM analysis (PPP-NCHAuD) were purchased from NANOSENSORS™, with resonance frequency in the range of 204-497 kHz and force constant in the range of 10-130. Acquired AFM images were analyzed using the JPK Data Processing software (acquisition processing). All data were collected using QI-mode.

Evaluation of oligomer hydrophobicity

LogP_{oct} Analysis. Octanol-water partition coefficients (LogP_{oct}) were calculated for oligomeric models (10-mers) in Materials Studio 2020, using an atom-based approach (ALogP method) for all molecular models containing C, H, N, O and S atoms.

Surface Area Analysis. Octanol-water partition coefficients (LogP_{oct}) were normalized by solvent accessible surface area (SA) using Materials Studio 2020. First, oligomers were subjected to a Geometry Optimization procedure using the Forcite Molecular Dynamics (MD) module with a COMPASS II force field. The force field contains information on important parameters, like preferred bond lengths, bond angles, torsion angles, partial charges, and van der Waals radii that influence the conformation. To minimize energy and determine a preferred conformation, these simulations ran until the energy of the oligomer decreased below predetermined convergence criteria (1×10^{-4} kcal mol^{-1} energy convergence, 0.005 kcal mol^{-1}/Å force convergence, and 5×10^{-5} Å displacement convergence). Second, these SA values represent solvent accessible surface area created by an algorithm that rolls a ball over the surface of the oligomer. To ensure the SA values are meaningful in the context of octanol-water partition coefficients (LogP_{oct}), the probe had a 1.4 Å radius to match the size of a water molecule.

Models. Scheme S1 depicts a representative example of PDEAEMA-based (**P1**) 10-mers betainised with 0, 10, 30, 50 and 100 mol% of each betainisation reagent (2-BES, 3-BPS, 4-BBS, or 3-CPS). Although the sulfobetaine side-groups varied depending on the betainisation reagent, blocky oligomers with a consistent *trans* conformation were selected for analysis in all cases.

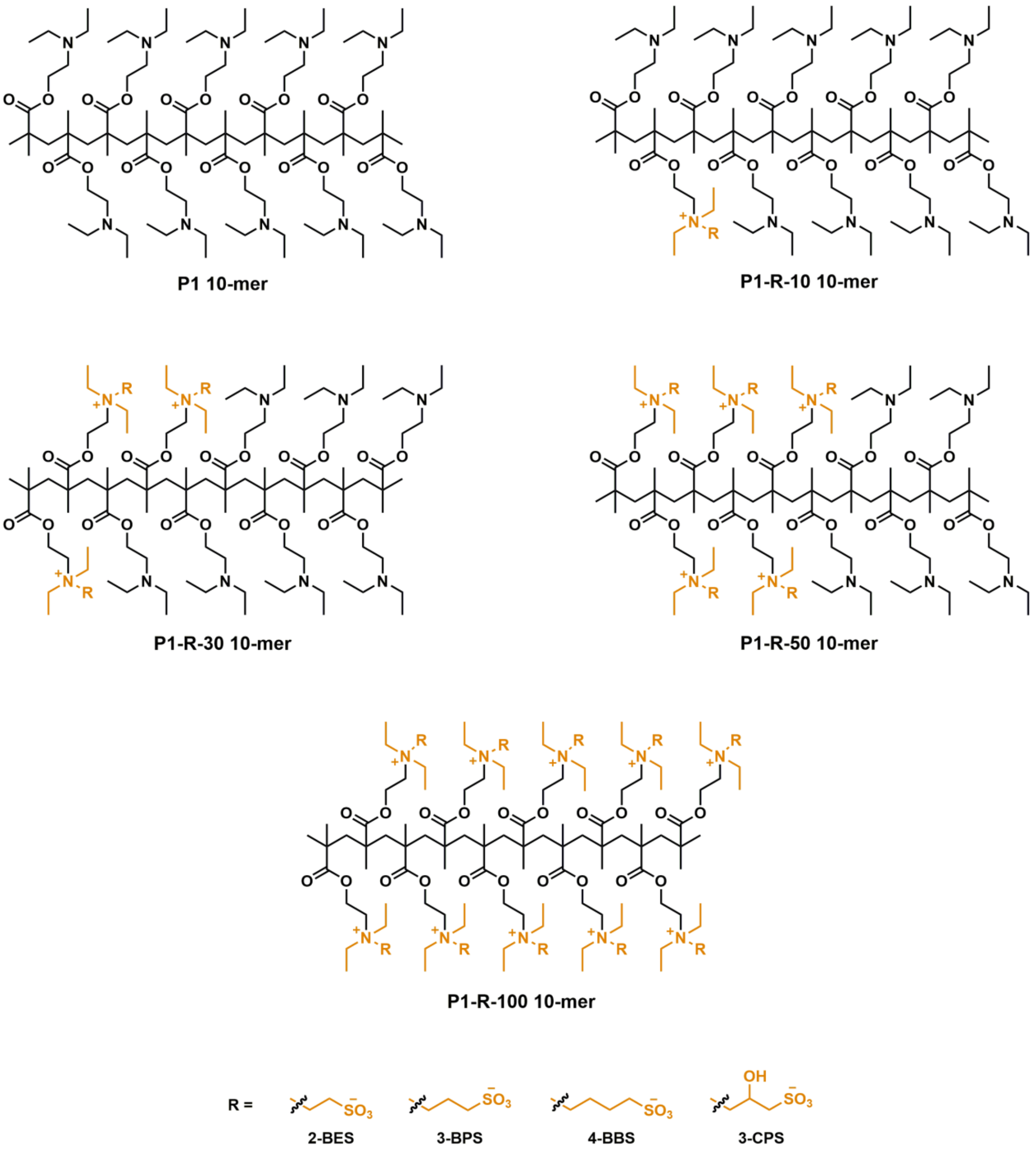

Scheme S1: Representative structures of PDEAEMA-based (**P1**) 10-mers betainised with 0, 10, 30, 50 and 100 mol% of each betainisation reagent (2-BES, 3-BPS, 4-BBS, or 3-CPS) used for LogP_{oct} calculations.

Characterization data for poly(*N*,*N*'-dimethyl(methacryloylethyl)ammonium propane sulfonate) (PDMAPS) macro-CTA

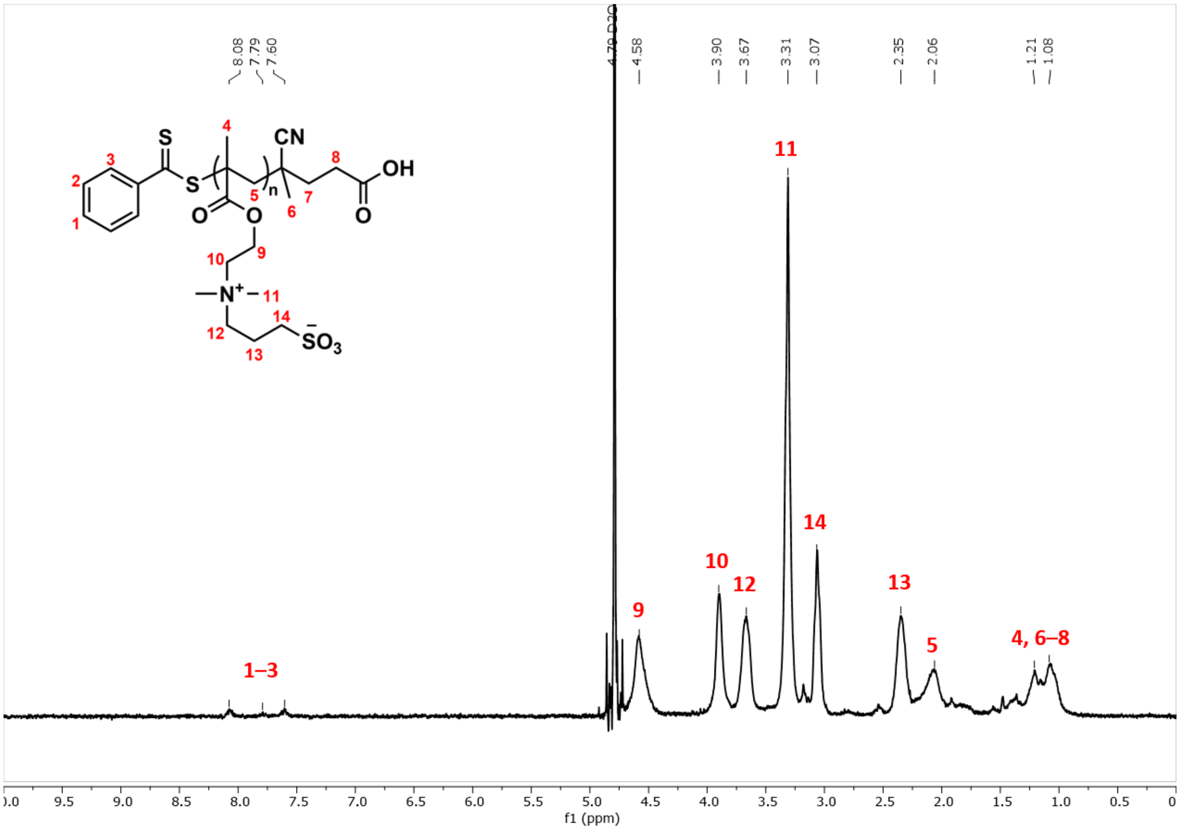

Figure S1: ^{1}H-NMR spectrum of poly(*N*,*N*'-dimethyl(methacryloylethyl)ammonium propane sulfonate) (PDMAPS) steric stabilizer in D_2O containing 0.5 M NaCl.

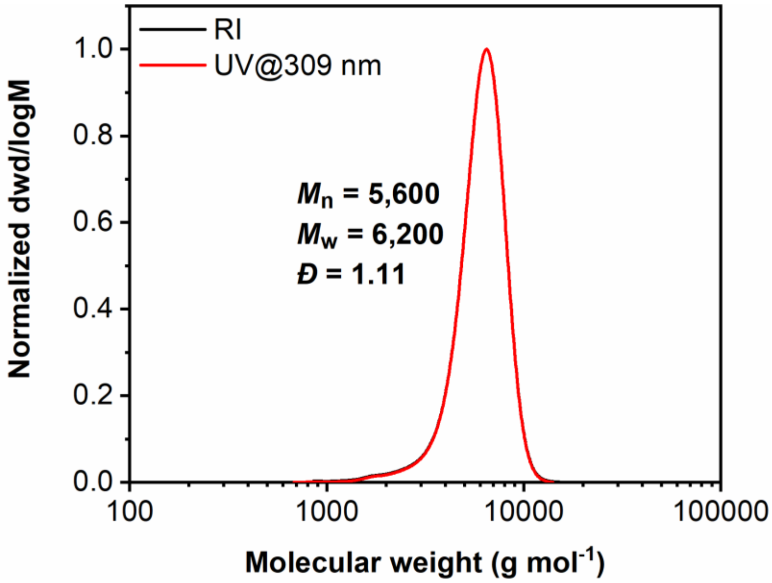

Figure S2: Normalized SEC RI (black trace) and SEC UV (red trace, λ = 309 nm) molecular weight distributions for PDMAPS macro-CTA, along with corresponding M_n, M_w, and *Đ* values calculated based on PEG standards using H_2O:MeOH (80:20) + 0.1 M $NaNO_3$ as the eluent.

Table S1: Molecular characteristics of PDMAPS macro-CTA, as determined by ^{1}H-NMR spectroscopy and SEC analysis.

[DMAPS]/[CPAD]	% Conv.^{a}	$M_{n,\ theo.}$ (kDa)^b	$M_{n,\ NMR}$ (kDa)^c	$M_{n,\ SEC}$ (kDa)^d	*Đ*, SEC^d
**18**	~90	4.8	5.2	5.6	1.11

^{a}Monomer conversion calculated from ^{1}H-NMR spectroscopy in D_2O containing 0.5 M NaCl. ^{b}Calculated from conversion. ^{c}Calculated using end-group analysis from ^{1}H-NMR spectroscopy. ^{d}M_n and *Đ* values calculated from PEG standards using H_2O:MeOH (80:20) + 0.1 M $NaNO_3$ as the eluent.

Supplementary characterization data for PDMAPS-*b*-P(DEAEMA-*co*-EGDMA) platform particles (P1)

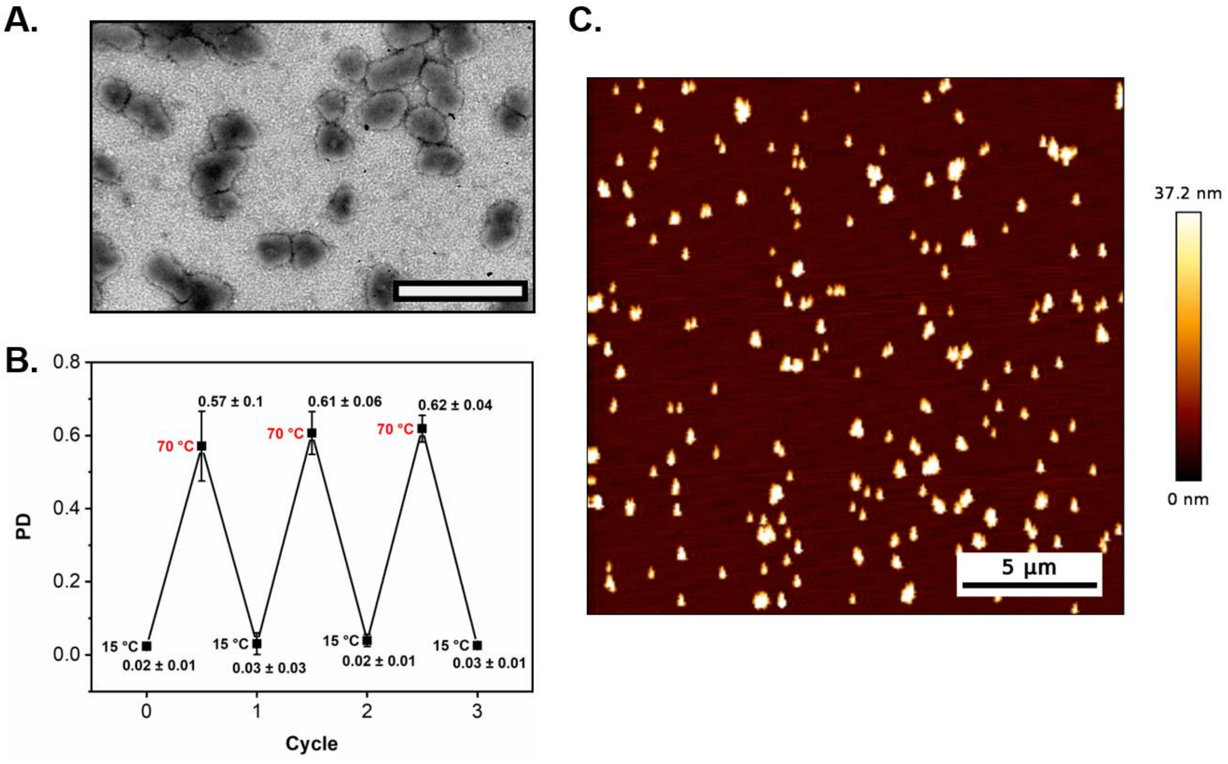

Figure S3: (A) Representative dry-state TEM image of PDEAEMA-based **P1** particles, stained with 1 wt% uranyl acetate (UA) solution (scale bar represents 500 nm). (B) Reversible aggregation behavior for **P1** particles reporting changes in PD as a function of solution temperature. Data was recorded over 3 heating–cooling cycles from 15–70 ºC in a single step of 55 ºC by variable temperature DLS analysis, run at a particle concentration of 1 mg mL^{-1} in 0.3 M NaCl solution at pH = 8.0. (C) Representative AFM height image of **P1** particles (scale bar represents 5 μm).

DLS analysis of PDEAEMA-based P1 particles betainised with 10, 30, 50 and 100 mol% 2-BES (P1-2-BES-10, 30, 50, 100)

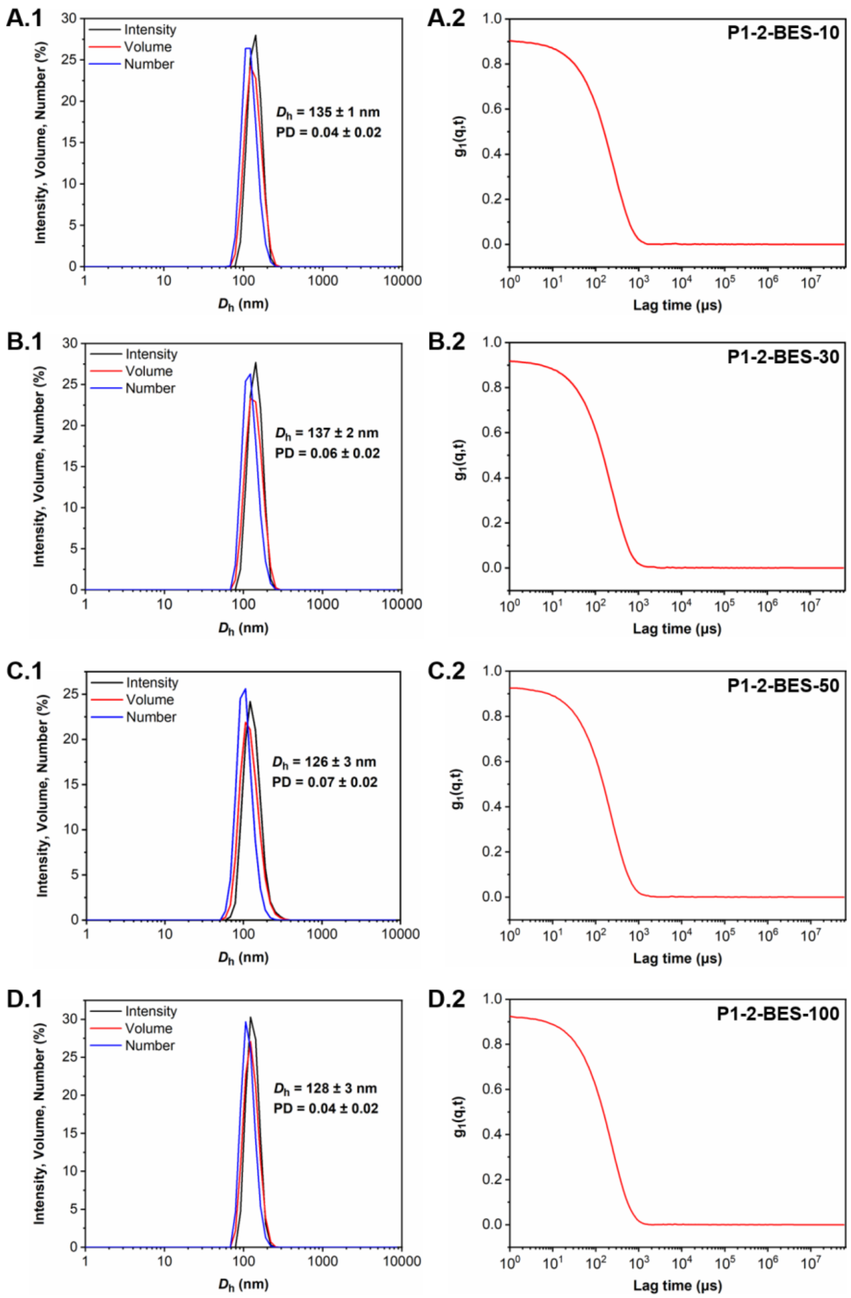

Figure S4: (1) Size distributions along with average D_h and PD values (from 4 repeat measurements) and (2) corresponding correlation function, obtained by DLS analysis, for **P1-2-BES-X** particles at (A) 10%, (B) 30%, (C) 50% and (D) 100% degree of betainisation. Recorded at 15 °C, conc. = 0.1 mg mL^{-1} in 0.3 M NaCl solution (pH = 8.0).

DLS analysis of PDEAEMA-based P1 particles betainised with 10, 30, 50 and 100 mol% 3-BPS (P1-3-BPS-10, 30, 50, 100)

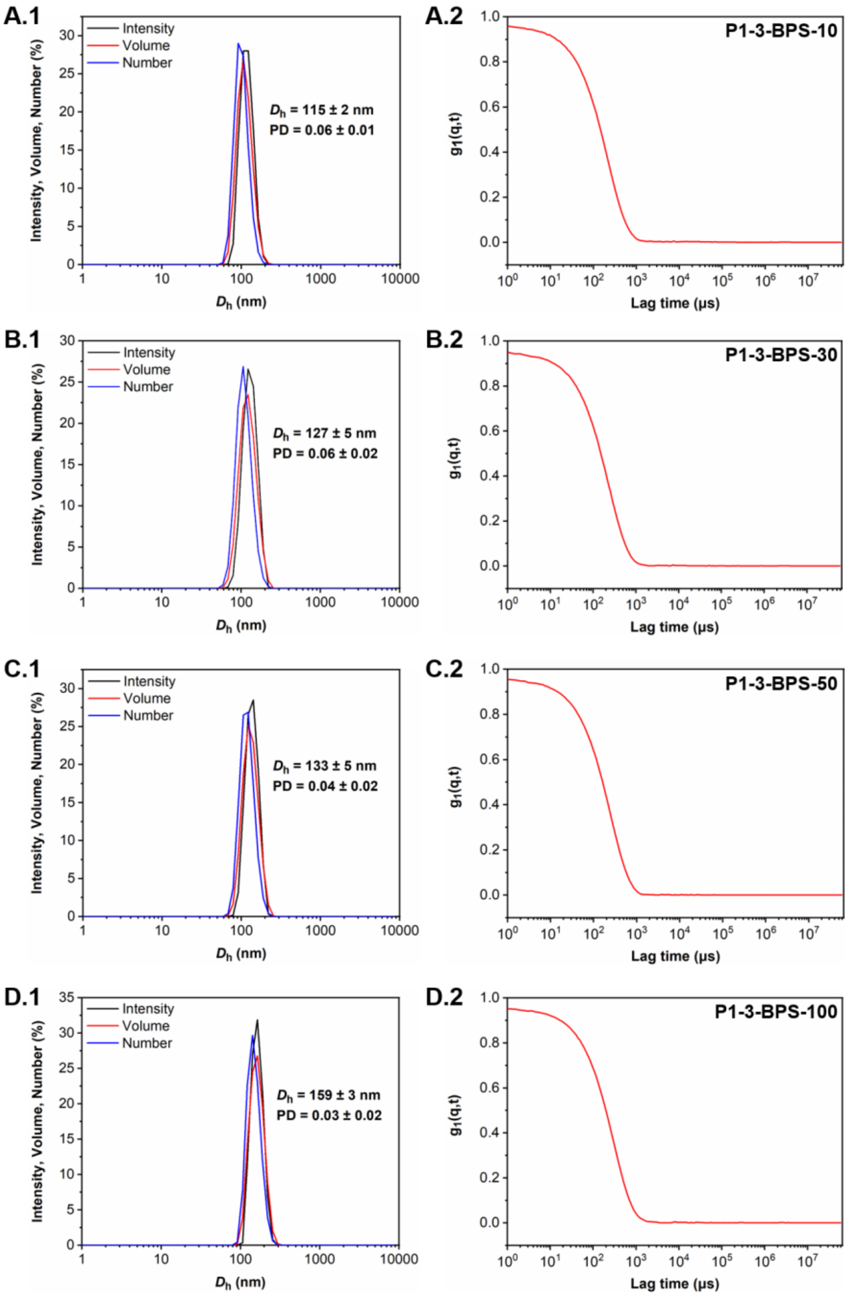

Figure S5: (1) Size distributions along with average D_h and PD values (from 4 repeat measurements) and (2) corresponding correlation function, obtained by DLS analysis, for **P1-3-BPS-X** particles at (A) 10%, (B) 30%, (C) 50% and (D) 100% degree of betainisation. Recorded at 15 °C, conc. = 0.1 mg mL^{-1} in 0.3 M NaCl solution (pH = 8.0).

DLS analysis of PDEAEMA-based P1 particles betainised with 10, 30, 50 and 100 mol% 4-BBS (P1-4-BBS-10, 30, 50, 100)

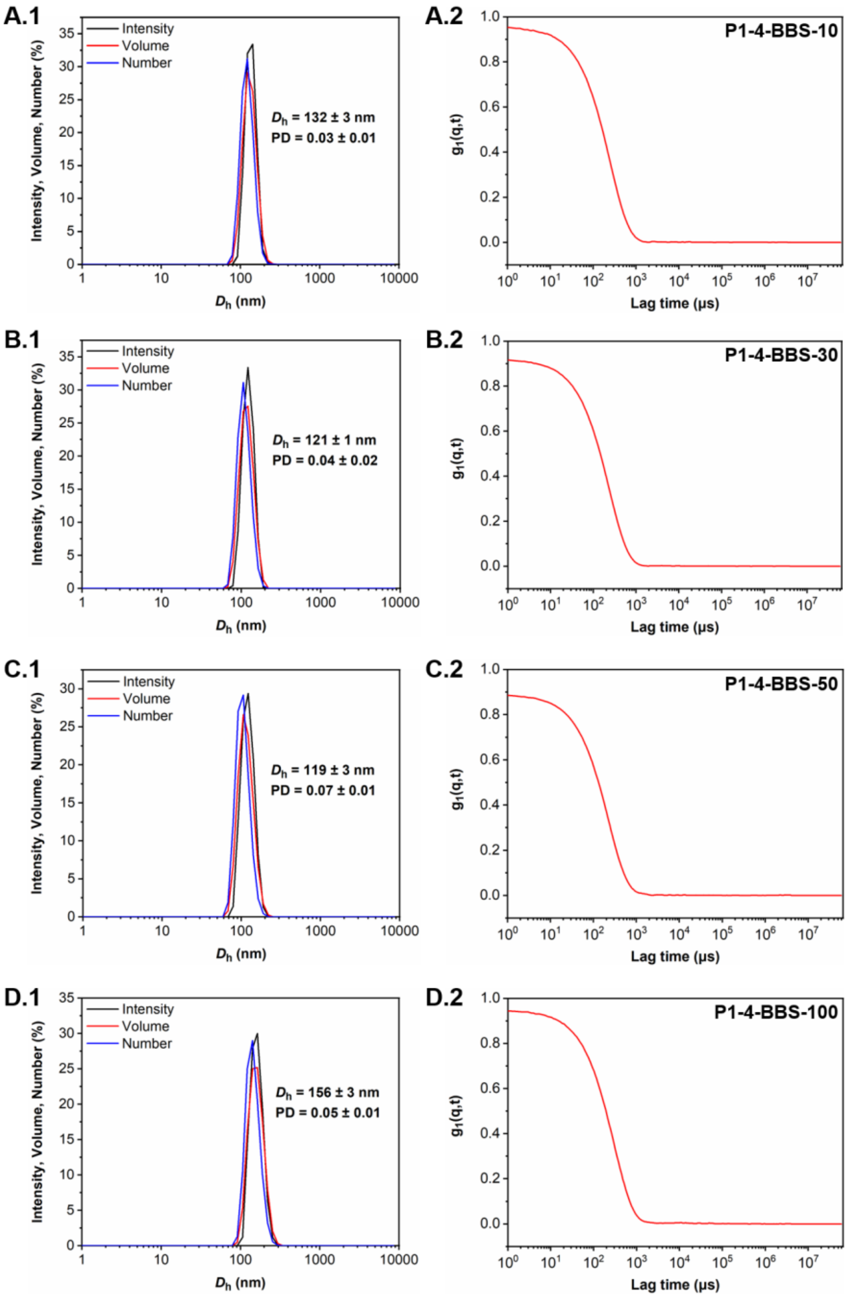

Figure S6: (1) Size distributions along with average D_h and PD values (from 4 repeat measurements) and (2) corresponding correlation function, obtained by DLS analysis, for **P1-4-BBS-X** particles at (A) 10%, (B) 30%, (C) 50% and (D) 100% degree of betainisation. Recorded at 15 °C, conc. = 0.1 mg mL^{-1} in 0.3 M NaCl solution (pH = 8.0).

DLS analysis of PDEAEMA-based P1 particles betainised with 10, 30, 50 and 100 mol% 3-CPS (P1-3-CPS-10, 30, 50, 100)

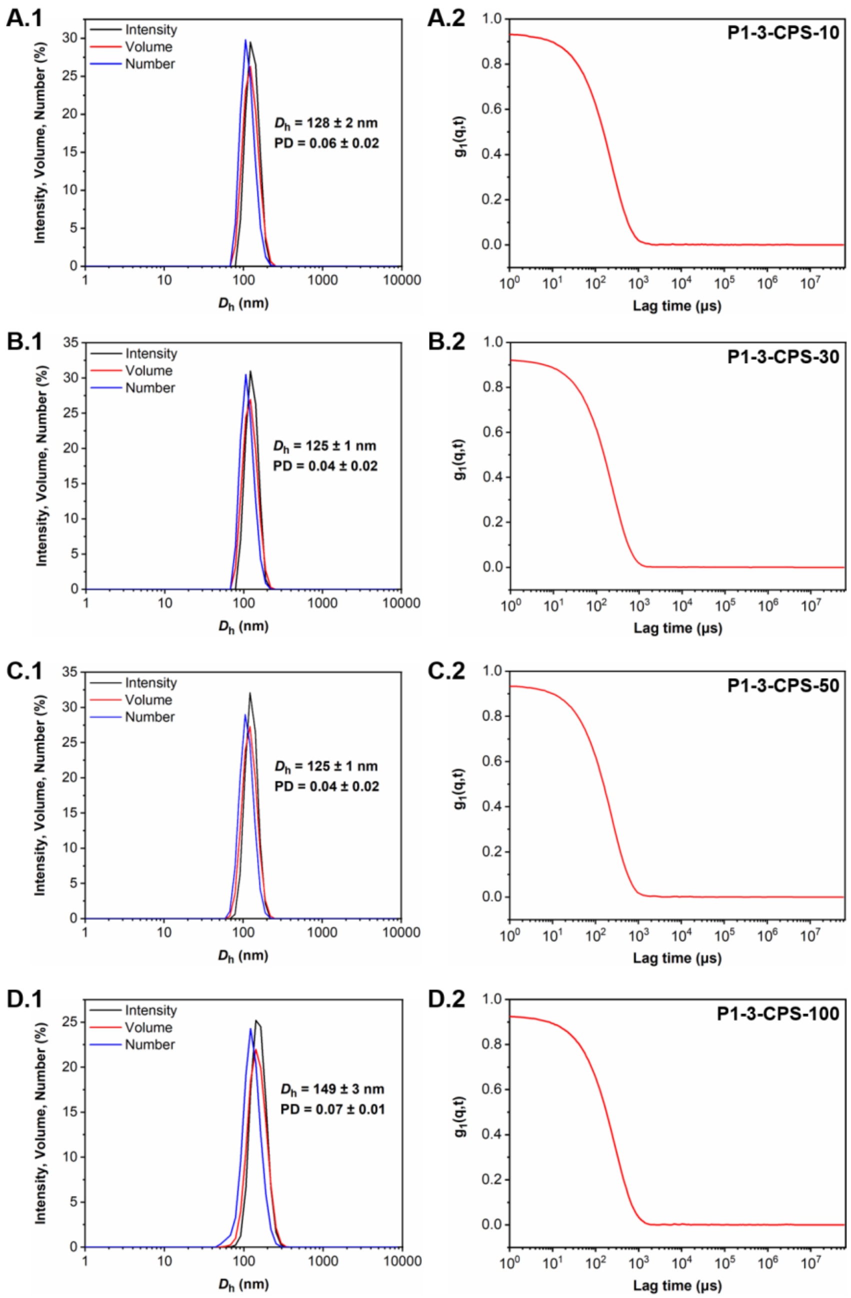

Figure S7: (1) Size distributions along with average D_h and PD values (from 4 repeat measurements) and (2) corresponding correlation function, obtained by DLS analysis, for **P1-3-CPS-X** particles at (A) 10%, (B) 30%, (C) 50% and (D) 100% degree of betainisation. Recorded at 15 °C, conc. = 0.1 mg mL^{-1} in 0.3 M NaCl solution (pH = 8.0).

Representative dry-state TEM and AFM images of P1 particles betainised with (A) 2-BES, (B) 3-BPS, (C) 4-BBS, and (D) 3-CPS at 30 mol% (P1-R-30)

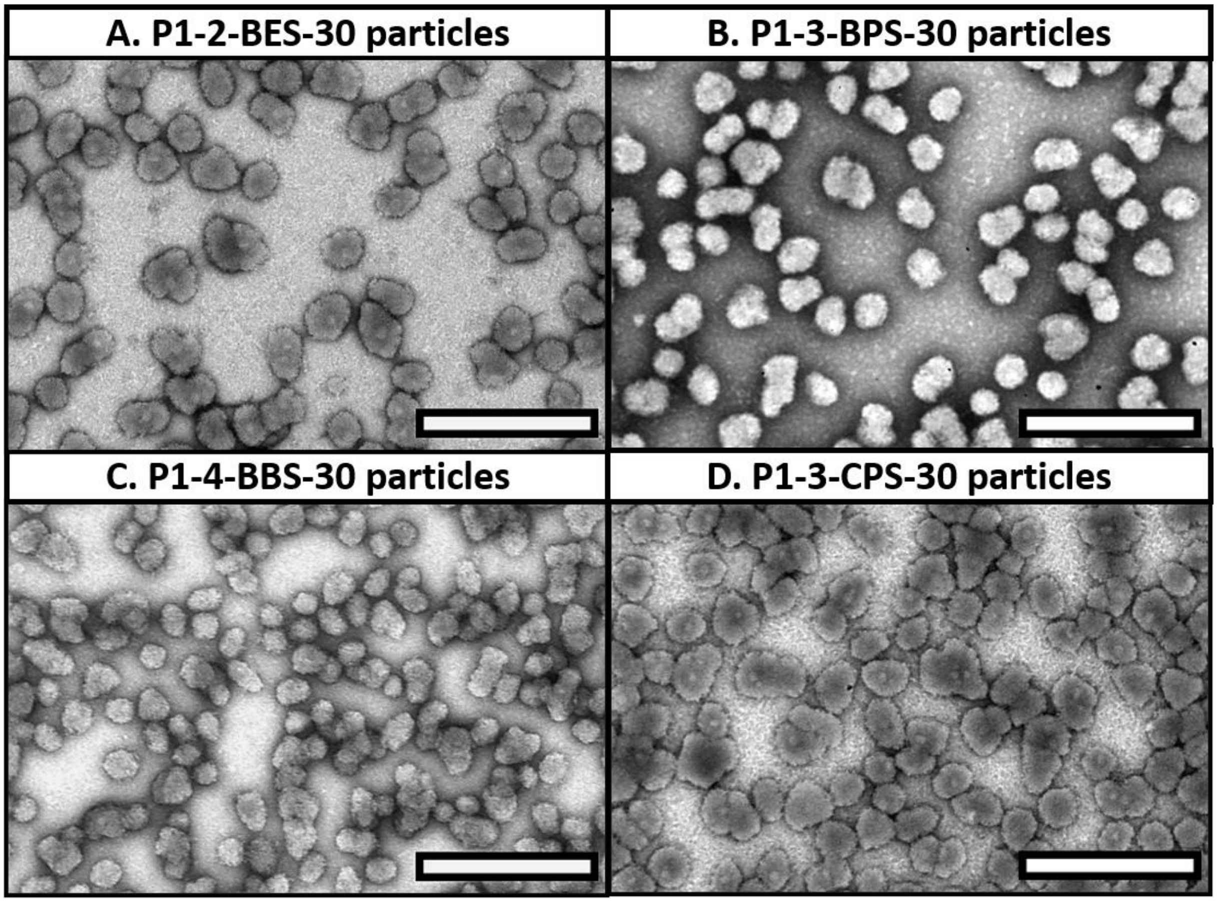

Figure S8: Representative dry-state TEM images of PDEAEMA-based **P1** particles betainised with (A) 2-BES, (B) 3-BPS, (C) 4-BBS, and (D) 3-CPS at 30 mol% (**P1-R-30**), stained with 1 wt% uranyl acetate (UA) solution (scale bar represents 500 nm).

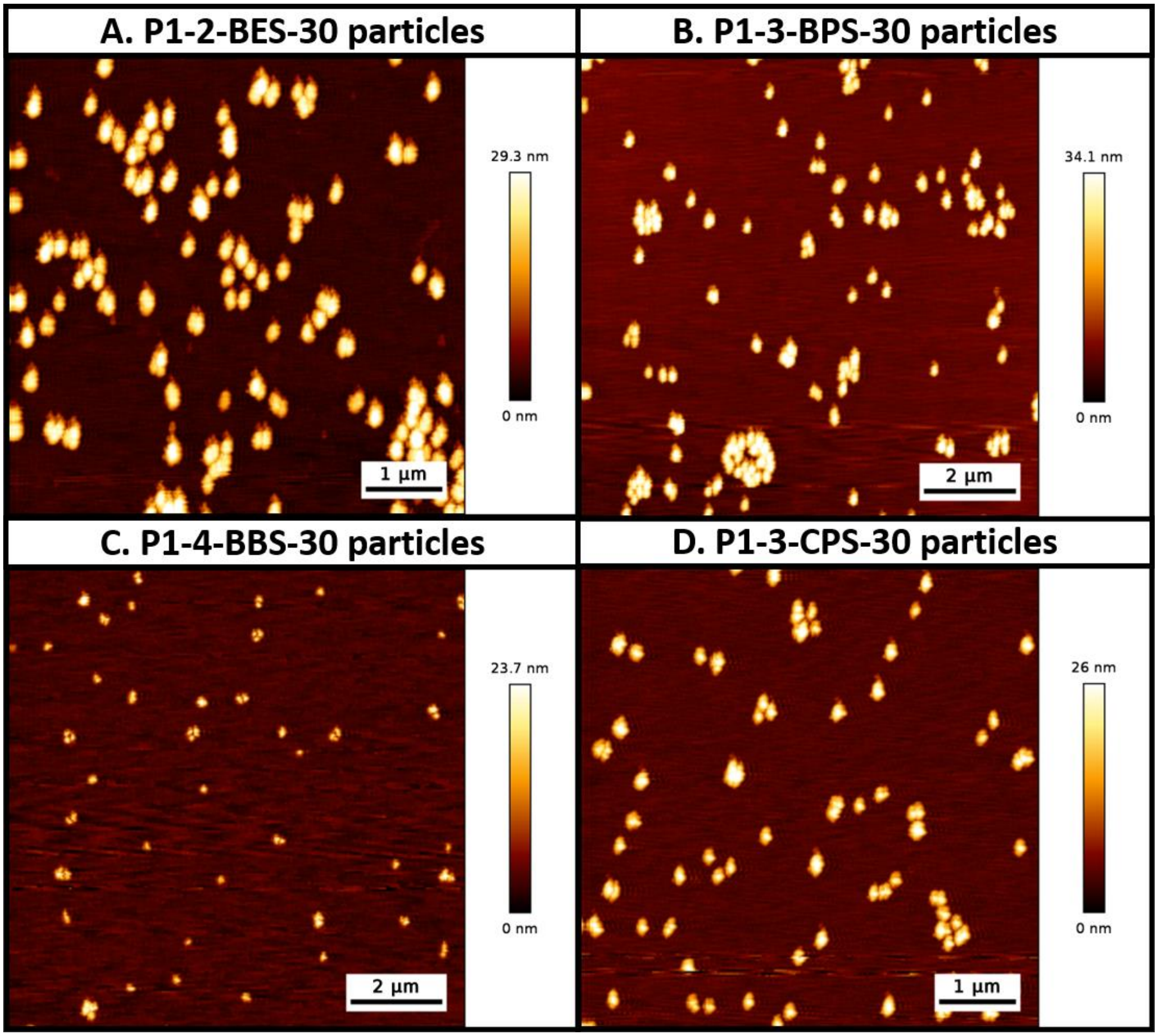

Figure S9: Representative AFM height images of PDEAEMA-based **P1** particles betainised with (A) 2-BES, (B) 3-BPS, (C) 4-BBS, and (D) 3-CPS at 30 mol% (**P1-R-30**).

Reversibility of the thermoresponsive behavior for the betainised P1-R particles

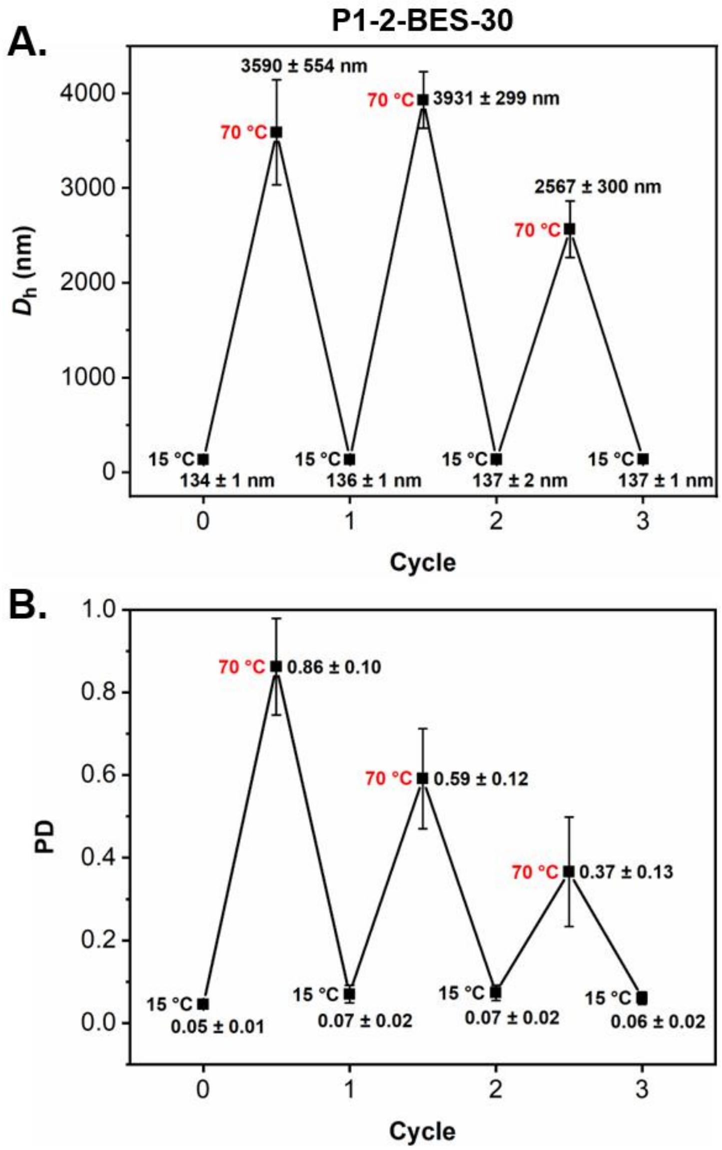

Figure S10: Reversible aggregation behavior for **P1-2-BES-30** particles reporting changes in (A) D_h and (B) PD as a function of solution temperature. Data was recorded over 3 heating–cooling cycles from 15–70 ºC in a single step of 55 ºC by variable temperature DLS analysis, run at a particle concentration of 1 mg mL^{-1} in 0.3 M NaCl solution (pH = 8.0).

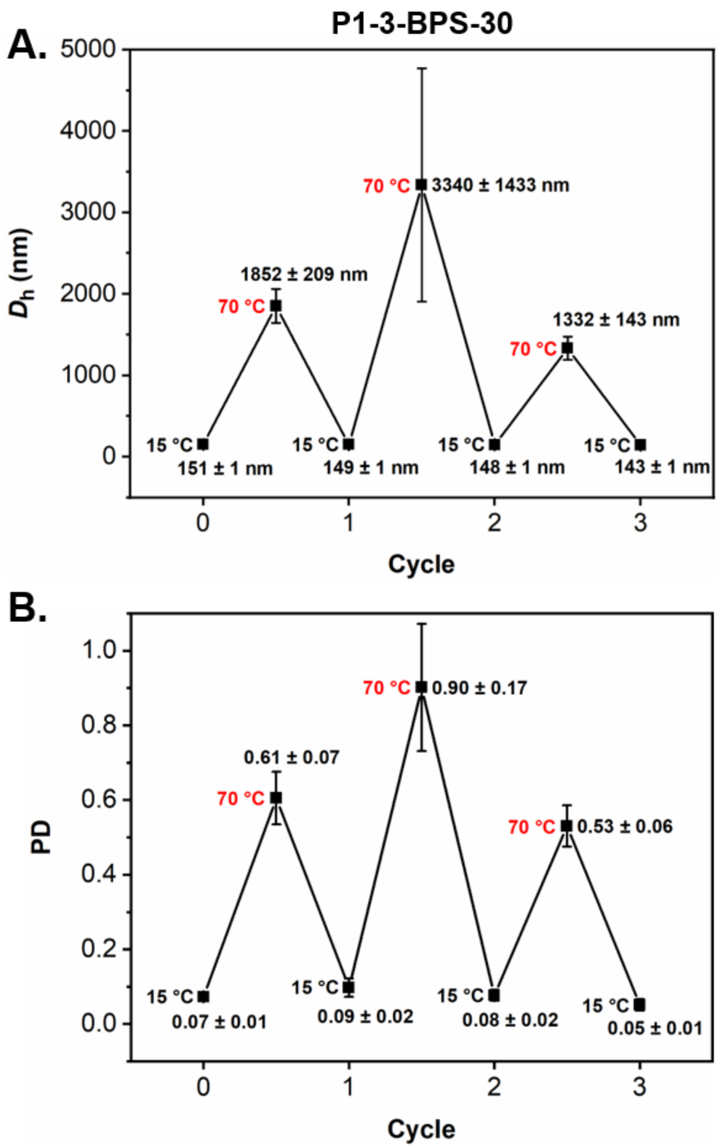

Figure S11: Reversible aggregation behavior for **P1-3-BPS-30** particles reporting changes in (A) D_h and (B) PD as a function of solution temperature. Data was recorded over 3 heating–cooling cycles from 15–70 ºC in a single step of 55 ºC by variable temperature DLS analysis, run at a particle concentration of 1 mg mL^{-1} in 0.3 M NaCl solution (pH = 8.0).

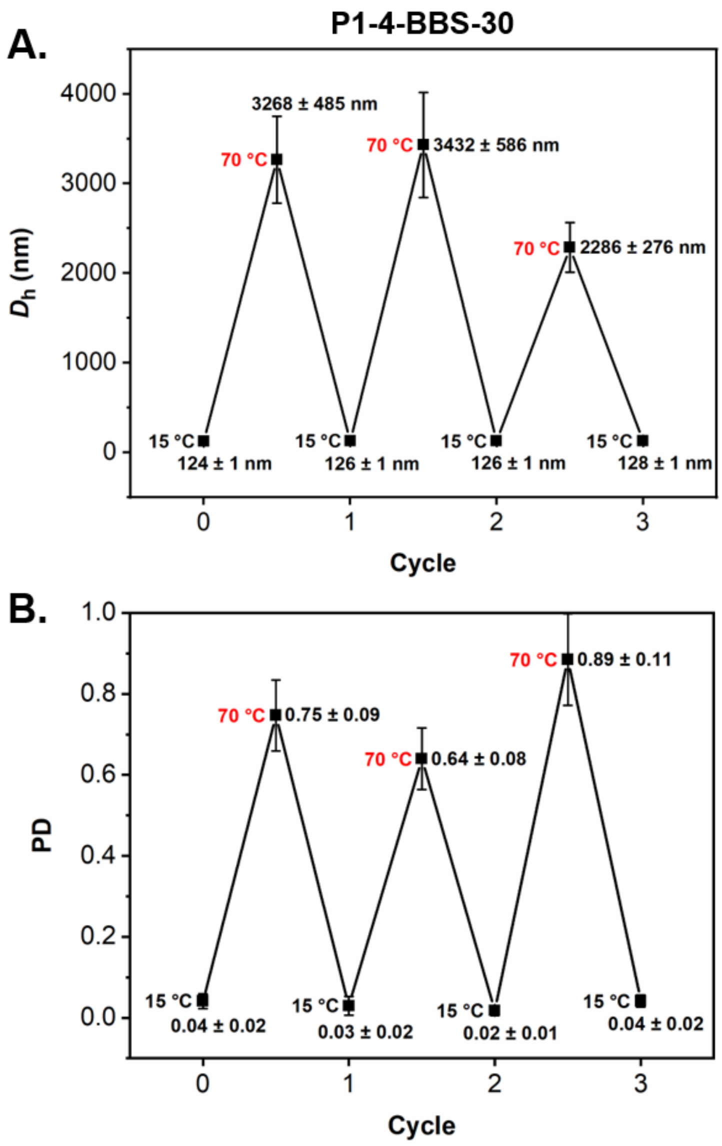

Figure S12: Reversible aggregation behavior for **P1-4-BBS-30** particles reporting changes in (A) D_h and (B) PD as a function of solution temperature. Data was recorded over 3 heating–cooling cycles from 15–70 ºC in a single step of 55 ºC by variable temperature DLS analysis, run at a particle concentration of 1 mg mL^{-1} in 0.3 M NaCl solution (pH = 8.0).

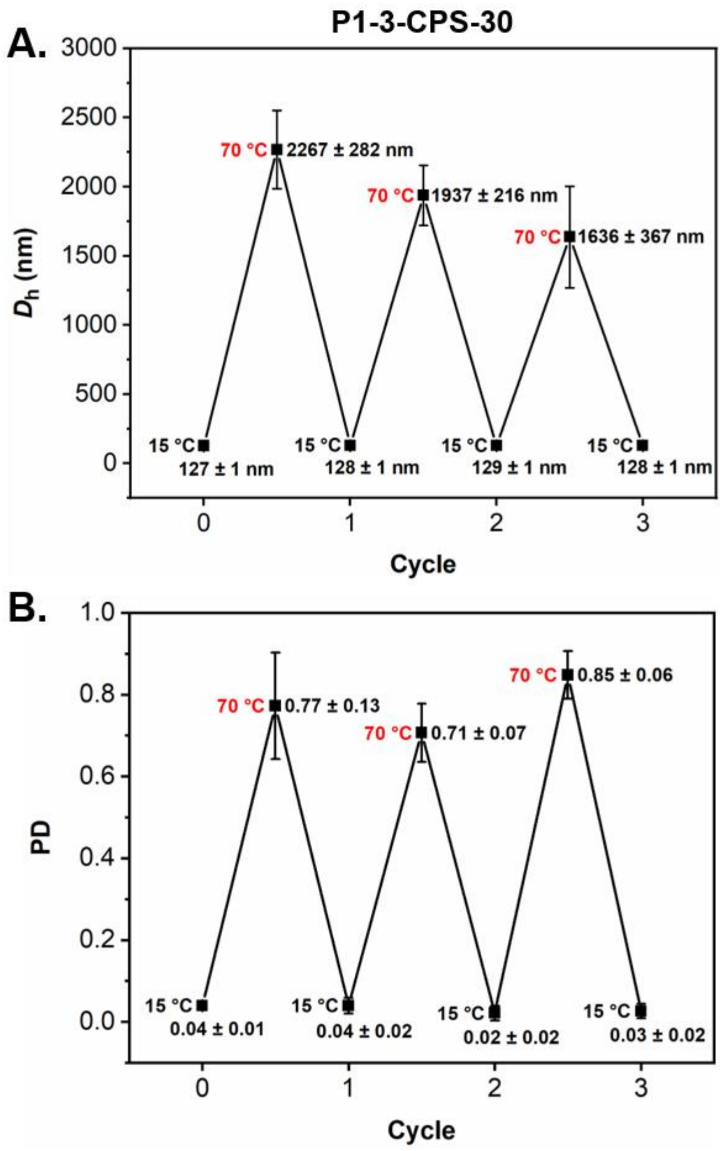

Figure S13: Reversible aggregation behavior for **P1-3-CPS-30** particles reporting changes in (A) D_h and (B) PD as a function of solution temperature. Data was recorded over 3 heating–cooling cycles from 15–70 ºC in a single step of 55 ºC by variable temperature DLS analysis, run at a particle concentration of 1 mg mL^{-1} in 0.3 M NaCl solution (pH = 8.0).

T_{CFT} calculations for the betainised P1-R particles by Piecewise linear fitting

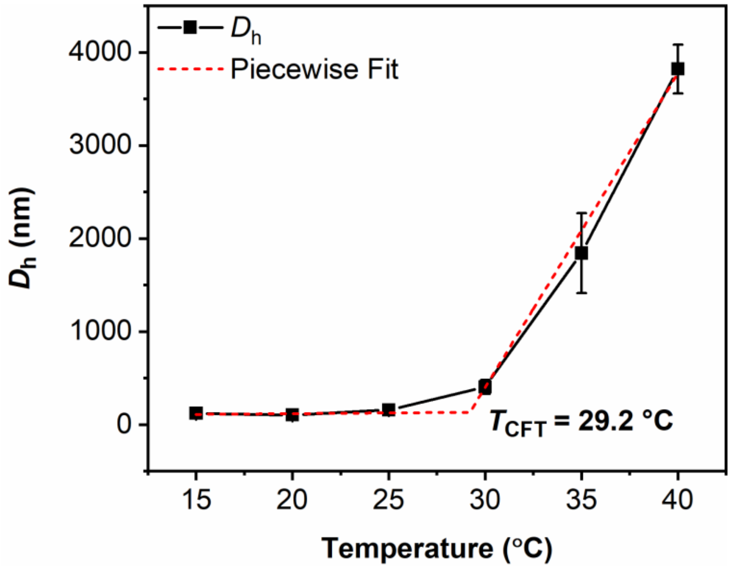

Figure S14: DLS temperature trend plot for PDEAEMA-based **P1** particles highlighting the Piecewise linear fitting application for determining T_{CFT}. Temperature trend was recorded from 15–90 ºC in steps of 5 ºC min^{-1} using a 1 mg mL^{-1} particle solution in 0.3 M NaCl (pH = 8.0).

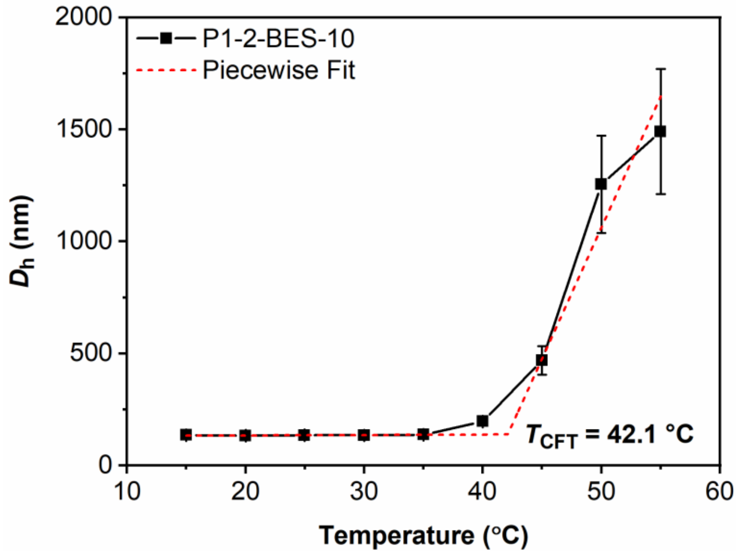

Figure S15: DLS temperature trend plot for **P1-2-BES-10** particles highlighting the Piecewise linear fitting application for determining T_{CFT}. Temperature trend was recorded from 15–90 ºC in steps of 5 ºC min^{-1} using a 1 mg mL^{-1} particle solution in 0.3 M NaCl (pH = 8.0).

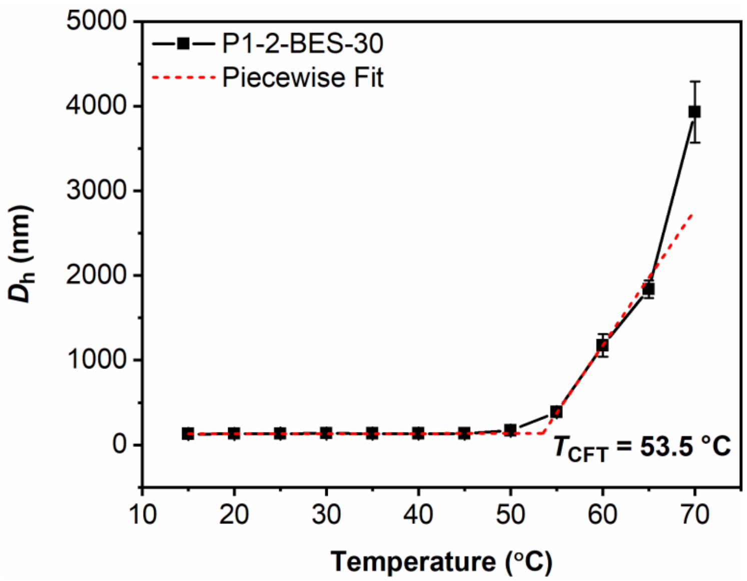

Figure S16: DLS temperature trend plot for **P1-2-BES-30** particles highlighting the Piecewise linear fitting application for determining T_{CFT}. Temperature trend was recorded from 15–90 ºC in steps of 5 ºC min^{-1} using a 1 mg mL^{-1} particle solution in 0.3 M NaCl (pH = 8.0).

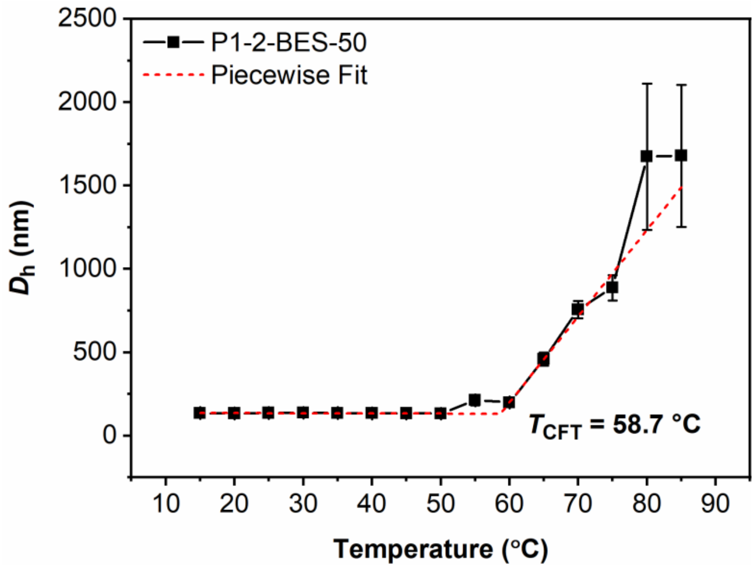

Figure S17: DLS temperature trend plot for **P1-2-BES-50** particles highlighting the Piecewise linear fitting application for determining T_{CFT}. Temperature trend was recorded from 15–90 ºC in steps of 5 ºC min^{-1} using a 1 mg mL^{-1} particle solution in 0.3 M NaCl (pH = 8.0).

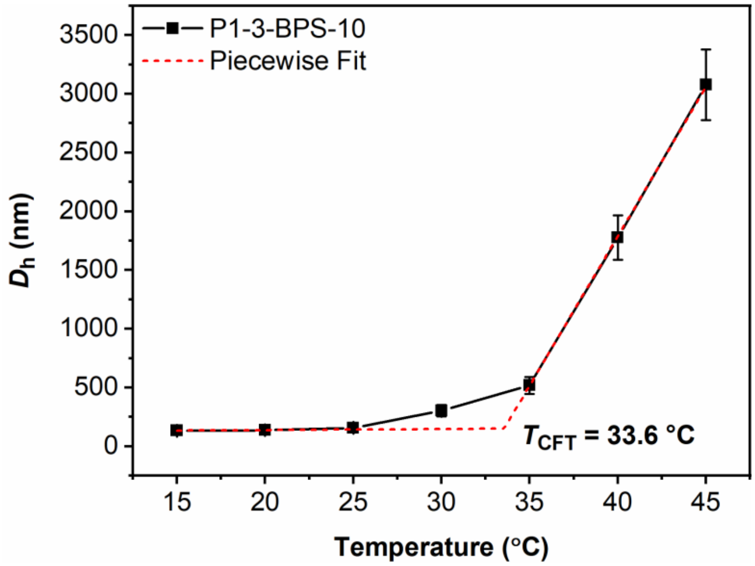

Figure S18: DLS temperature trend plot for **P1-3-BPS-10** particles highlighting the Piecewise linear fitting application for determining T_{CFT}. Temperature trend was recorded from 15–90 ºC in steps of 5 ºC min^{-1} using a 1 mg mL^{-1} particle solution in 0.3 M NaCl (pH = 8.0).

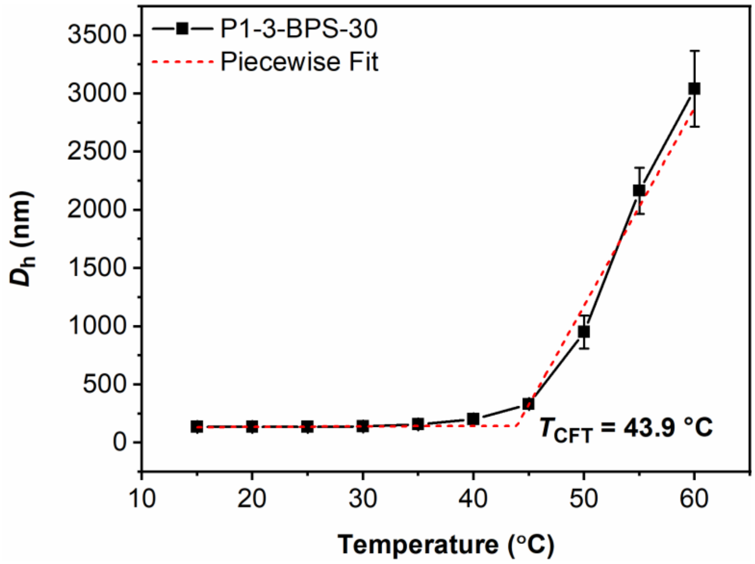

Figure S19: DLS temperature trend plot for **P1-3-BPS-30** particles highlighting the Piecewise linear fitting application for determining T_{CFT}. Temperature trend was recorded from 15–90 ºC in steps of 5 ºC min^{-1} using a 1 mg mL^{-1} particle solution in 0.3 M NaCl (pH = 8.0).

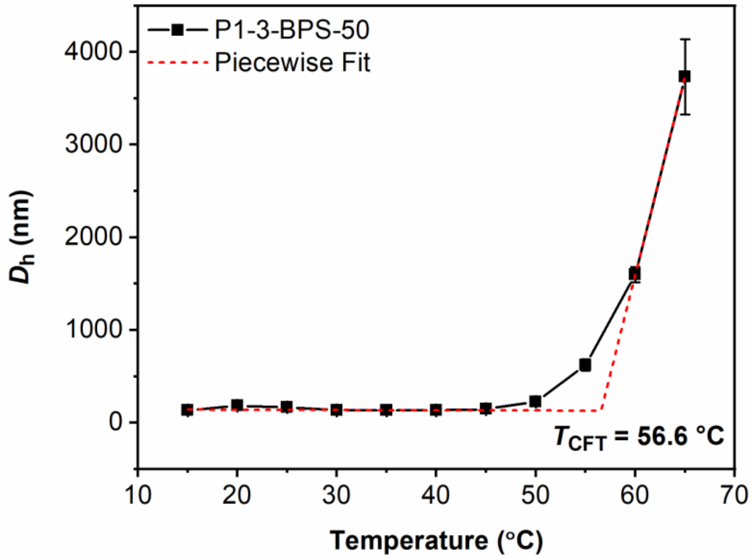

Figure S20: DLS temperature trend plot for **P1-3-BPS-50** particles highlighting the Piecewise linear fitting application for determining T_{CFT}. Temperature trend was recorded from 15–90 ºC in steps of 5 ºC min^{-1} using a 1 mg mL^{-1} particle solution in 0.3 M NaCl (pH = 8.0).

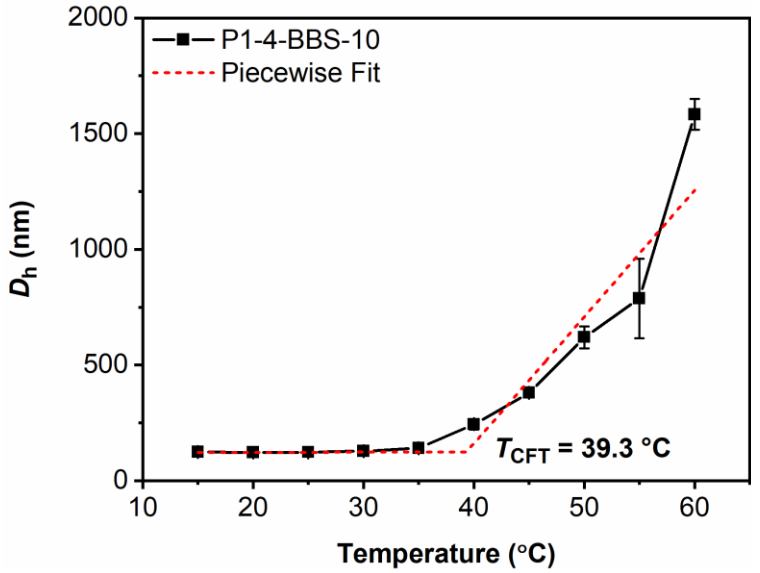

Figure S21: DLS temperature trend plot for **P1-4-BBS-10** particles highlighting the Piecewise linear fitting application for determining T_{CFT}. Temperature trend was recorded from 15–90 ºC in steps of 5 ºC min^{-1} using a 1 mg mL^{-1} particle solution in 0.3 M NaCl (pH = 8.0).

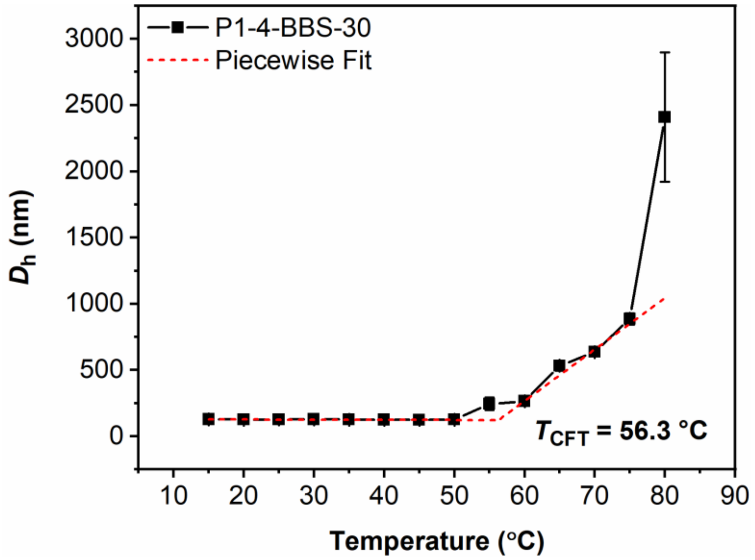

Figure S22: DLS temperature trend plot for **P1-4-BBS-30** particles highlighting the Piecewise linear fitting application for determining T_{CFT}. Temperature trend was recorded from 15–90 ºC in steps of 5 ºC min^{-1} using a 1 mg mL^{-1} particle solution in 0.3 M NaCl (pH = 8.0).

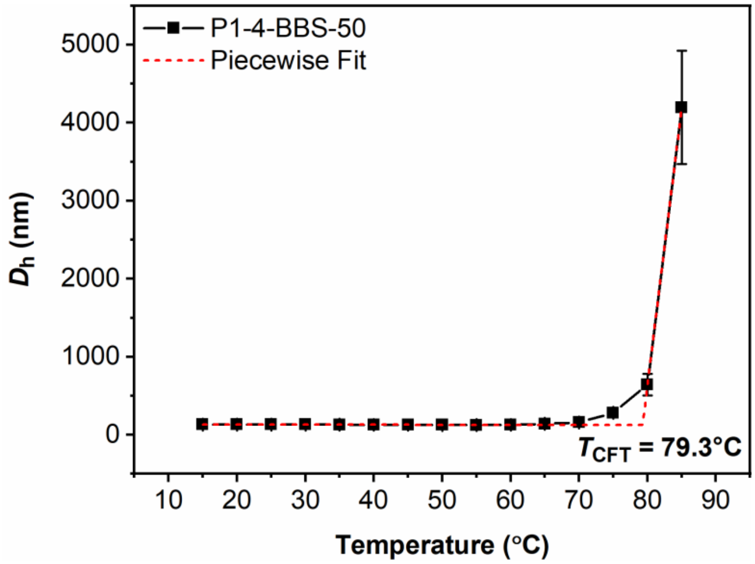

Figure S23: DLS temperature trend plot for **P1-4-BBS-50** particles highlighting the Piecewise linear fitting application for determining T_{CFT}. Temperature trend was recorded from 15–90 ºC in steps of 5 ºC min^{-1} using a 1 mg mL^{-1} particle solution in 0.3 M NaCl (pH = 8.0).

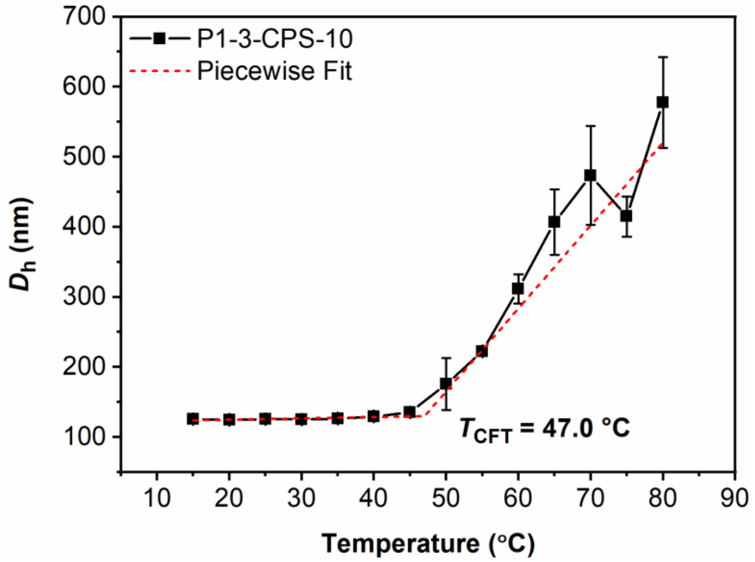

Figure S24: DLS temperature trend plot for **P1-3-CPS-10** particles highlighting the Piecewise linear fitting application for determining T_{CFT}. Temperature trend was recorded from 15–90 ºC in steps of 5 ºC min^{-1} using a 1 mg mL^{-1} particle solution in 0.3 M NaCl (pH = 8.0).

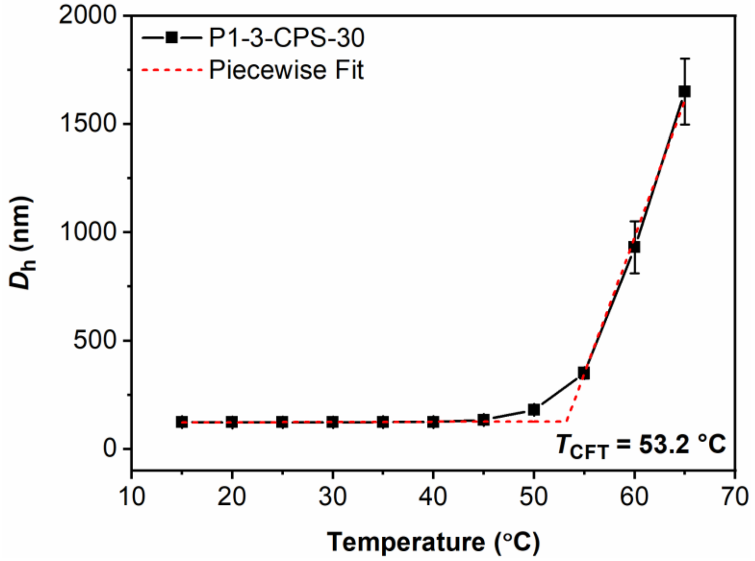

Figure S25: DLS temperature trend plot for **P1-3-CPS-30** particles highlighting the Piecewise linear fitting application for determining T_{CFT}. Temperature trend was recorded from 15–90 ºC in steps of 5 ºC min^{-1} using a 1 mg mL^{-1} particle solution in 0.3 M NaCl (pH = 8.0).

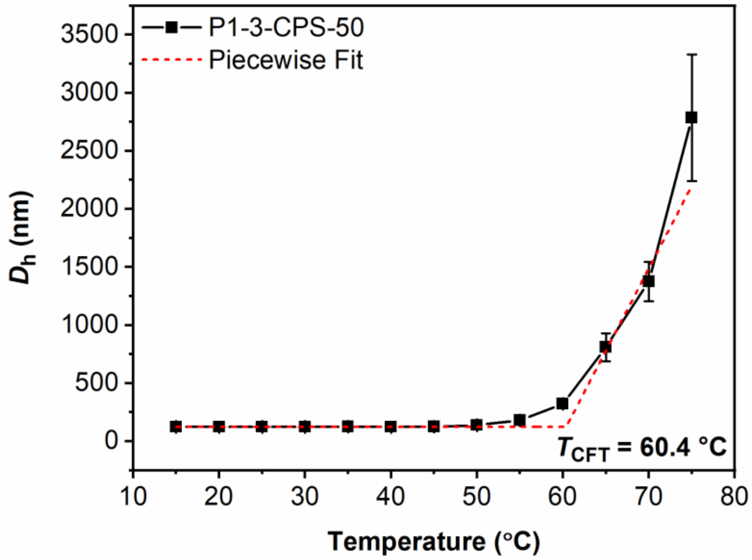

Figure S26: DLS temperature trend plot for **P1-3-CPS-50** particles highlighting the Piecewise linear fitting application for determining T_{CFT}. Temperature trend was recorded from 15–90 ºC in steps of 5 ºC min^{-1} using a 1 mg mL^{-1} particle solution in 0.3 M NaCl (pH = 8.0).

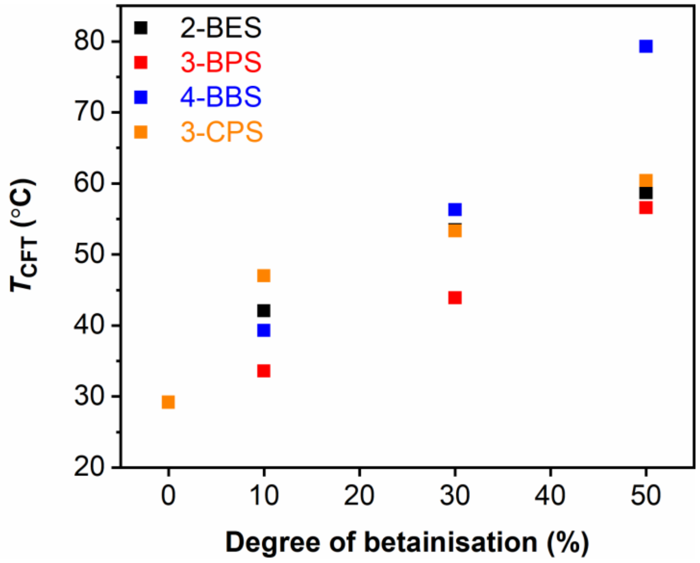

Figure S27: Correlation plot for measured T_{CFT} values as a function of the degree of betainisation for each betainisation reagent used.

Table S2: Linear fitting analysis reporting the slope and Pearson's correlation coefficient (PCC) for measured T_{CP} values as a function of degree of betainisation for each one of the betainisation reagents utilized.

Betainisation Reagent	T_{CP} Slope	T_{CP} PCC
**2-BES**	0.10	0.81
**3-BPS**	0.10	0.81
**4-BBS**	0.08	0.82
**3-CPS**	0.45	0.89
